# Exploring factors related to changes in body composition, insulin sensitivity and aerobic capacity in response to a 12-week exercise intervention in overweight and obese women with and without polycystic ovary syndrome

**DOI:** 10.1371/journal.pone.0182412

**Published:** 2017-08-03

**Authors:** David Scott, Cheryce L. Harrison, Samantha Hutchison, Barbora de Courten, Nigel K. Stepto

**Affiliations:** 1 Department of Medicine, School of Clinical Sciences at Monash Health, Faculty of Medicine, Nursing and Health Sciences, Monash University, Clayton, Victoria, Australia; 2 Department of Medicine – Western Campus and Australian Institute for Musculoskeletal Science, The University of Melbourne, St Albans, Victoria, Australia; 3 Monash Centre for Health Research and Implementation, School of Public Health and Preventive Medicine, Monash University, Clayton, Victoria, Australia; 4 Diabetes and Vascular Medicine Unit, Monash Health, Clayton, Victoria, Australia; 5 Institute of Sport Exercise and Active Living (ISEAL) Victoria University, Melbourne, Victoria, Australia; Texas A&M University, UNITED STATES

## Abstract

**Objective:**

To determine factors associated with differential changes in body fat, insulin resistance and aerobic capacity following a 12-week exercise intervention in overweight and obese women with and without polycystic ovary syndrome (PCOS).

**Methods:**

16 overweight and obese women (9 PCOS; 7 without PCOS) completed a supervised progressive 12-week exercise program. Primary outcomes included changes in indicators of insulin sensitivity (including glucose infusion rate relative to fat-free mass [GIR/FFM]), body composition, and aerobic capacity (VO_2_ peak; 12 participants only). Comparisons were made between women with and without PCOS, and between participants who lost ≥5% (classified as exercise responders) and <5% (non-responders) in body fat (assessed by dual-energy X-ray absorptiometry).

**Results:**

Training decreased body fat percentage by (mean; 95% CI) -2.3%; -5.3, 0.7% in women with PCOS and by -6.4%; -10.9, -1.9% in women without PCOS (P = 0.08). Ten women (7 PCOS; 3 without PCOS) did not reduce body fat by ≥5%. All participants improved VO_2_ peak (mean change 27%; 16–39%) but four (2 PCOS; 2 without PCOS) demonstrated decreases in GIR/FFM (mean change for whole cohort: 37%; 3–71%). Android-gynoid fat ratio (0.58; 0.51, 0.66 vs 0.46; 0.40, 0.51; P<0.01) was significantly higher and GIR/FFM (6.69; 3.49, 9.90 vs 11.44; 9.15, 13.72 mg/kg/min; P = 0.01) was significantly lower in non-responders compared with responders at baseline, but non-responders had significant post-training decreases in android-gynoid ratio (-0.02; -0.04, -0.01; P = 0.03), and increases in VO_2_ peak (7.24; 2.28, 12.21 mL/kg/min; P = 0.01) and GIR/FFM (1.44; 0.27, 2.61 mg/kg/min; P = 0.02). In women with PCOS, pre-training VO_2_ peak was significantly negatively correlated with change in total body fat (r = -0.75; P = 0.02), and pre-training fasting glucose negatively correlated with changes in VO_2_ peak (r = -0.76; P = 0.04), but positively correlated with changes in GIR (r = 0.67; P = 0.046).

**Conclusion:**

A high proportion of overweight and obese women with PCOS had small reductions in body fat following a 12-week exercise intervention, but nevertheless significantly reduced relative central adiposity and improved aerobic capacity and insulin sensitivity.

## Introduction

Polycystic ovary syndrome (PCOS) affects up to one in five reproductive-aged women in Australia and is associated with reproductive dysfunction, as well as metabolic consequences including obesity, type 2 diabetes and cardiovascular disease [1]. Given the known benefits of exercise targeting weight loss for preventing and reversing cardiometabolic conditions, exercise is recommended as an important therapy for women with PCOS [2, 3]. However, we previously reported that a 12-week moderate to vigorous intensity exercise program failed to normalise insulin sensitivity in overweight women with PCOS compared to overweight women without PCOS [4]. Women with PCOS have also been observed to have significantly smaller increases in growth hormone in response to exercise than controls [5].

Poor exercise responsiveness appears highly heritable [6] and may affect around 10% of exercise participants in the general population [7]. However, modifiable factors, including higher body fat and insulin resistance are common in women with PCOS [8, 9], and may increase risk for poor exercise responsiveness in this population. It has been reported that 15–20% of individuals with type 2 diabetes [10], and as many as 40% of middle-aged individuals at high risk of type 2 diabetes [11], experience minimal improvements in metabolic health following exercise. Furthermore, higher total body fat mass is associated with smaller improvements in physical function following an exercise intervention, irrespective of exercise adherence in overweight and obese older adults [12]. It has been observed in overweight postmenopausal women however that aerobic capacity significantly increases in response to exercise even in those who do not reduce fat mass substantially [13]. Thus, although PCOS women tend to have higher amounts of body fat [14], it is unclear whether this may also attenuate exercise benefits and whether differences in body fat distribution are more important than overall body fat for exercise responsiveness.

The aims of this secondary analysis of a 12-week exercise intervention in overweight women with and without PCOS to determine factors associated with differential changes in body fat, insulin resistance and aerobic capacity following a 12-week exercise intervention in overweight and obese women with and without PCOS.

## Material and methods

### Study design and participants

This study was a secondary analysis of a 12-week exercise intervention for overweight and obese (BMI >25 kg/m^2^) and inactive (<100 minutes per week of self-reported moderate and vigorous physical activity) women. Participants constituted a subset of a cross-sectional study of pre-menopausal women with and without PCOS [4, 9, 15] and were recruited through community advertisements. The Monash Health Research Advisory and Ethics Committee approved the study and participants gave written informed consent. All investigations were conducted according to the principles expressed in the Declaration of Helsinki. The trial was registered previously (ISRCTN84763265).

**Fig 1:**
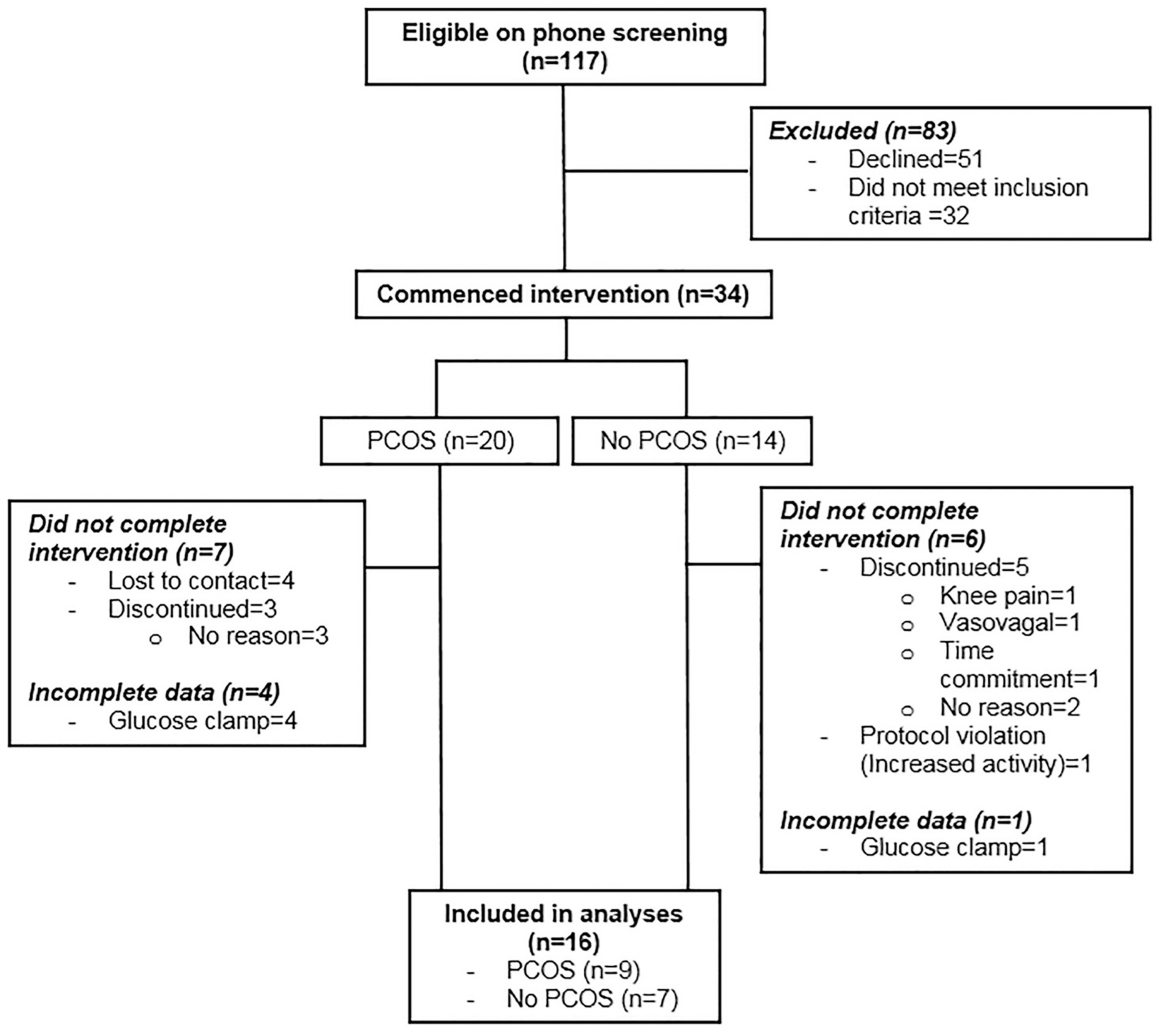
Consolidated Standards of Reporting Trials (CONSORT) flowchart describing progress through the study.

As described in Fig 1, 117 women were initially screened of whom 34 (n = 20 with PCOS; n = 14 without PCOS) were eligible and consented to the exercise intervention. Thirteen PCOS and eight non-PCOS women completed the study, and amongst these 16 (9 with PCOS and 7 without PCOS) had complete body composition and insulin sensitivity data for this secondary analysis. Diagnosis of PCOS was undertaken by endocrinologists (S.K.H.) based on meeting NIH criteria with demonstrating irregular menstrual cycles (21 or 35 days) and clinical (hirsutism, acne) or biochemical (elevation of at least one circulating ovarian androgen) hyperandrogenism. As these women were not assessed for polycystic ovaries on ultrasound, they still meet the Rotterdam criteria but exact phenotype was unknown [16]. Hyperprolactinemia, thyroid dysfunction and specific adrenal disorders were excluded clinically and where indicated, biochemically. All women without PCOS had regular menses and no evidence of clinical or biochemical hyperandrogenism. Exclusion criteria were age <20 or >40 years, lipid-lowering agents, smoking, diabetes, recent weight change of 5 kilograms or more in the previous six months, actively trying to lose weight and pregnancy.

At screening, standard diet and lifestyle advice were delivered based on recommendations provided by the National Heart Foundation of Australia (www.heartfoundation.com.au) [17]. Medications known to affect end-points, including insulin sensitisers, anti- androgens and hormonal contraceptives were ceased three-months prior to baseline. All data were collected in the follicular phase for women without PCOS in order to minimise potential impact of female reproductive hormones on study outcomes. However, as women with PCOS had irregular menses they were measured at times convenient to participants.

### Exercise intervention

Participants undertook 12 weeks of supervised, progressive, moderate and vigorous exercise training on a motorised treadmill [4, 15, 18]. Participants attended three 45–60 min sessions supervised by study staff each week, which sequentially alternated between moderate-intensity (20–60 minutes of walking or jogging at 75–80% of maximal heart rate [HR_max_]) and high-intensity interval training (six to eight five-minute intervals at ∼95–100% HR_max_ with two-minute passive recovery periods). Participants progressed to eight repetitions by week four and reduced recovery time to 1 minute by week eight. Target exercise heart rates were achieved by altering speed and incline on the treadmill according to individual fitness [15]. Exercise adherence was assessed by recording the number of sessions attended, and indicators of exercise compliance were recorded, including total duration of exercise, total distance covered and average heart rate assessed by heart rate monitors (Polar Electro Oy, Kempele, Finland).

### Measurements

All measurements were obtained by study staff blinded to the PCOS status of participants. Body weight and height were assessed and body mass index (BMI) calculated as weight (kilograms) divided by height (metres) squared. Total body fat mass and fat-free mass, and android and gynoid, fat mass were estimated by a whole-body dual energy X-ray absorptiometry (DXA) scan (GE Lunar Prodigy, GE Lunar Corp, Madison WI, USA; operating system version 9). VO_2_ peak was assessed during a graded exercise test (GXT) on a motorised treadmill (Biodex RTM 500, New York, USA) treadmill until volitional fatigue. Expired gases were collected and analysed during the GXT using the MOXUS modular system (AEI Technologies, Pittsburgh, PA, USA). The Moxus was calibrated prior to each test with known gases (16% O2; 4% CO2; BOC gases Australia) and volume (3L Hans Rudolph syringe).

The primary outcome was insulin sensitivity as measured by glucose infusion rate (mg/m^2^/min) assessed by the euglycaemic–hyperinsulinaemic clamp technique as previously described [15]. Clamp timing was standardised to 48 h after exercise and following a standardised high-carbohydrate diet for 72 hours before an overnight fast. Insulin (Actrapid; Novo Nordisk, Bagsvaerd, Denmark) was infused at 40 mU/m^2^ min for 120 min, with plasma glucose maintained at ∼5 mmol/L using variable infusion rates of 25% glucose. Glucose infusion rate (GIR) was calculated during steady state, achieved in the last 30 minutes of the clamp and expressed as glucose (mg) per kg of fat free mass per min (GIR/FFM). Stored blood samples (-80°C) were batch analysed for plasma fasting glucose and insulin, homeostatic model assessment of insulin resistance (HOMA-IR) was calculated [9].

### Statistical analyses

Prior to analyses, data were assessed for normality by Shapiro-Wilk tests stratified by PCOS status and fasting insulin and HOMA-IR were log transformed. Independent samples t-tests compared changes in total body fat and VO_2_ peak from pre- to post-training in women with and without PCOS. Independent samples t-tests and paired t-tests compared pre- and post-exercise values for body composition, VO_2_ peak, and indicators of insulin resistance and exercise compliance in women defined as exercise ‘responders’ (≥5% decrease in total body fat) versus ‘non-responders’ (<5% decrease in total body fat) following training [13], and a chi-square test compared proportions with and without PCOS. Pearson correlations stratified by PCOS status examined associations of pre-training participant characteristics with post-training changes in body composition, VO_2_ peak and indicators of insulin resistance. All statistical analyses were performed using SPSS version 23.0 (IBM, USA) and P-values <0.05 were considered statistically significant.

## Results

**Fig 2:**
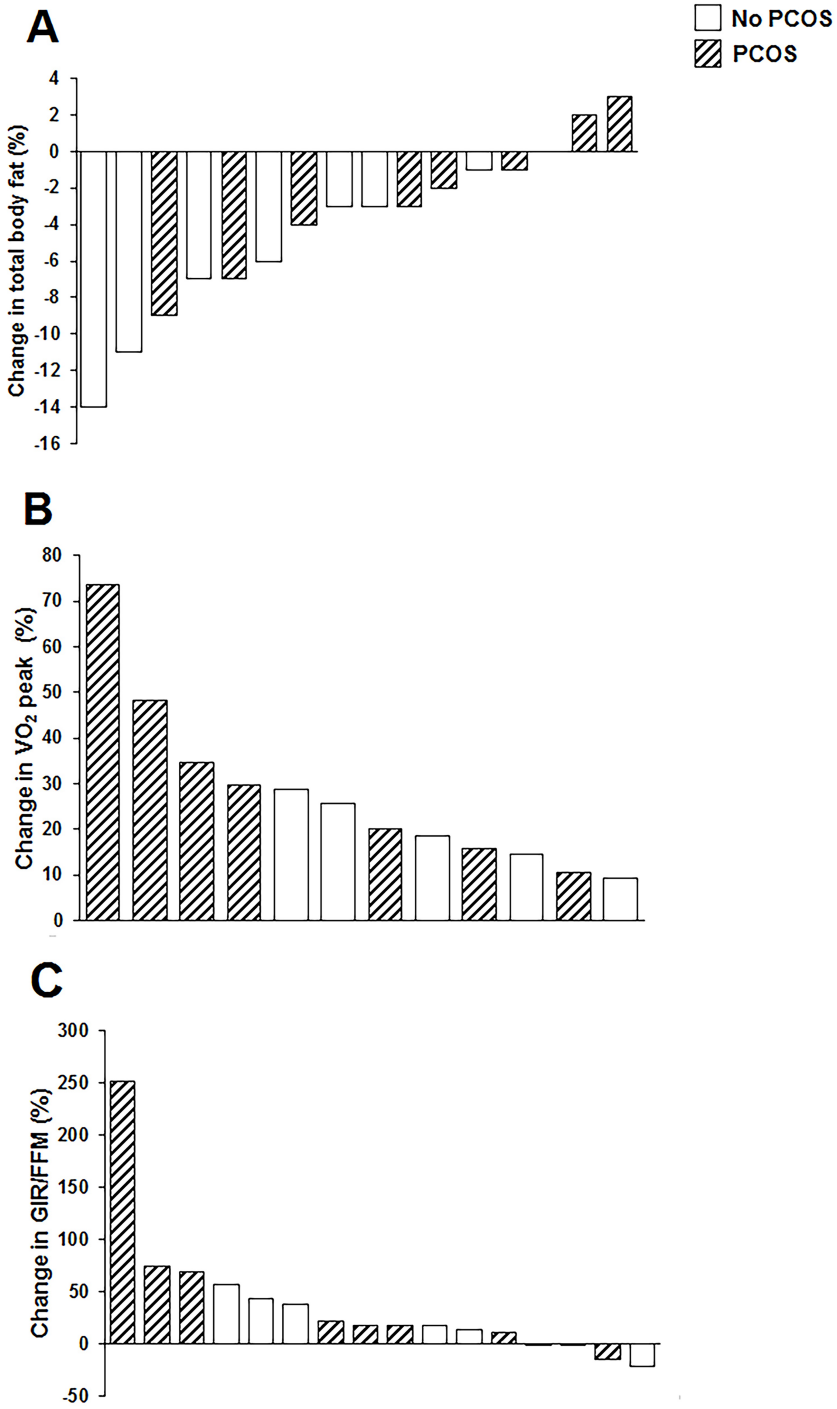
Individual participant changes in total body fat (A), VO_2_ peak (B) and GIR/FFM (C) from pre- to post-training according to PCOS status.

Compared with the 18 participants who were excluded due to non-completion or missing data (Table 1), the 16 included participants were of similar age (mean; 95% CI) (32.1 years; 29.4, 34.8 vs 32.1 years; 28.9, 35.3; P = 0.99), BMI (34.9 kg/m^2^; 31.9, 37.9 vs 36.29 kg/m^2^; 32.9, 39.4; P = 0.55), and GIR/FFM (9.39; 7.02, 11.76 mg/kg/min vs 8.47; 6.13, 10.81 mg/kg/min; P = 0.56). The majority of excluded participants were lost to contact (could not be contacted by phone or email; 4) or refused to continue (due to time commitment; 1, vasovagal episode; 1, knee soreness; 1, or no reason given; 5) in the exercise intervention by week six of the intervention. One participant who completed the intervention was excluded due to a protocol violation (commenced significant sustained physical activity outside the intervention), and five had incomplete GIR/FFM data at baseline or follow-up (Fig 1). Amongst the included participants, four (3 with PCOS, 1 without PCOS) did not have exercise tests either at baseline or follow-up and so were excluded from analyses of change in VO_2_ peak. As demonstrated by the body fat, VO_2_ peak and GIR/FFM changes reported in Fig 2, there was a wide range of individual responses to the exercise program. Ten (7 with PCOS and 3 without PCOS; P = 0.2) participants did not reduce their body fat by ≥5%, and were classified as non-responders. Training decreased body fat percentage by -2.3%; -5.3, 0.7% in women with PCOS and by -6.4%; -10.9, -1.9% in women without PCOS (P-value for difference between groups = 0.08). Participants with PCOS lost less gynoid fat compared with participants without PCOS (-0.02; -0.29, 0.24 kg vs -0.62; -1.14, -0.09 kg; P = 0.02). Conversely, all participants improved VO_2_ peak (27%; 16–39%) with no significant differences between women with PCOS and those without (P = 0.21). Four (2 with PCOS; 2 without PCOS) participants demonstrated no improvement in GIR/FFM (mean change for whole cohort: 37%; 3–71%) with no significant difference between groups (P = 0.39).

**Table 1:** Comparison of pre-training and post-training values for body composition and cardiometabolic parameters according to exercise responsiveness.

	Pre-training		Change to post-training	
	Non-Responders (N = 10)	Responders (N = 6)	Non-Responders; *P-value for change from pre-training*	Responders; *P-value for change from pre-training*
Age (y)	32.6 (28.6, 36.7)	31.2 (23.8, 38.6)	-	-
PCOS (%)*	70.0	33.3	-	-
BMI (kg/m^2^)	36.5 (31.7, 41.4)	35.5 (30.0, 41.0)	-0.46 (-1.03, 0.11); *0*.*10*	-0.97 (-2.34, 0.40); *0*.*1*
Fat-free mass (kg)	50.8 (45.0, 56.5)	48.4 (44.9, 51.9)	-0.67 (-1.67, 0.33); *0*.*16*	2.00 (0.99, 3.01); ***0*.*01***
Total fat mass (kg)	47.6 (39.3, 55.9)	47.0 (34.4, 59.7)	-0.48 (-1.20, 0.23); *0*.*16*	-4.48 (-7.32, -1.65); ***0*.*01***
Android fat (kg)	4.8 (3.8, 5.8)	4.0 (3.1, 4.9)	-0.17 (-0.33, 0.01); *0*.*05*	-0.40 (-0.60, -0.21); ***0*.*01***
Gynoid fat (kg)	8.2 (7.1, 9.3)	8.8 (6.4, 11.1)	-0.02 (-0.22, 0.21); *0*.*98*	-0.75 (-1.30, -0.20); ***0*.*02***
Android/gynoid ratio	0.58 (0.51, 0.66)	0.46 (0.40, 0.51)†	-0.02 (-0.04, -0.01); ***0*.*03***	-0.01 (-0.03, 0.01); *0*.*32*
VO_2_ peak (mL/kg/min); n = 12	24.5 (20.0, 29.1)	28.2 (20.5, 35.9)	7.24 (2.28, 12.21); ***0*.*01*** (N = 6)	5.95 (3.47, 8.43); ***0*.*01*** (N = 6)
Glucose (mmol/L)	5.0 (4.6, 5.3)	4.8 (4.5, 5.0)	0.00 (-0.27, 0.27); *1*.*0*	-0.10 (-0.43, 0.23); *0*.*47*
Log Insulin	1.4 (1.2, 1.5)	1.2 (1.1, 1,3)	-0.03 (-0.14, 0.07); *0*.*46*	0.01 (-0.32, 0.34); *0*.*93*
Log HOMA-IR	0.7 (0.5, 0.9)	0.5 (0.4, 0.7)	-0.03 (-0.15, 0.08); *0*.*51*	0.5 01 (-0.33, 0.34); *0*.*98*
GIR/FFM (mg/kg/min)	6.7 (3.5, 9.9)	11.4 (9.2, 13.7)†	1.44 (0.27, 2.61); ***0*.*02***	1.57 (-1.44, 4.57); *0*.*239*

All data are mean (95% CI) unless otherwise specified.

*Chi-square test

^†^Significantly different from non-responders (P = 0.01).

Baseline participant characteristics for exercise responders and non-responders defined according to body fat changes are presented in Table 1. There were no differences in mean age or PCOS status between groups. There were also no differences in total body fat, android and gynoid fat mass, however android-gynoid ratio was significantly higher in non-responders. Measures of insulin sensitivity were generally similar but GIR/FFM was significantly lower in non-responders at baseline. Table 1 also presents post-training values for body composition and indicators of insulin sensitivity according to exercise responsiveness. Total body fat, android fat and gynoid fat were significantly decreased post-training in responders, although the decrease in android fat in non-responders was of borderline significance (P = 0.05). Non-responders had a significant decrease in android-gynoid ratio post-training. Both responders and non-responders demonstrated a significant increase in VO_2_ peak, while only non-responders had significant increases in insulin sensitivity as measured by GIR.

As demonstrated in Table 2, there were no differences between responders and non-responders for exercise compliance or indicators of exercise workload. Women with PCOS covered significantly higher mean distance than women without PCOS for the duration of the intervention, however exercise workload (calculated as heart rate divided by distance covered (bpm/km), was significantly higher in women without PCOS.

**Table 2:** Comparison of indicators of exercise adherence and compliance during the 12-week exercise intervention according to exercise responsiveness and PCOS status.

	Non-Responders (N = 10)	Responders (N = 6)	P-value for difference between groups	PCOS (N = 9)	No PCOS (N = 7)	P-value for difference between groups
Total exercise sessions	32 (30, 33)	31 (29, 33)	0.79	32 (30, 34)	31 (29, 33)	0.74
Total exercise time (hrs)	29.7 (27.9, 31.5)	29.4 (26.2, 32.5)	0.82	30.1 (27.8, 32.5)	28.9 (27.1, 30.7)	0.37
Total exercise distance (km)	165.0 (28.0, 302.0)	77.0 (1.8, 155.9)	0.31	199.2 (55.7, 342.7)	46.1 (3.1, 89.0)	**0.04**
Mean exercise heart rate (bpm)	153 (146, 160)	156 (150, 162)	0.45	153 (145, 161)	156 (152, 159)	0.49
Mean heart rate multiplied by exercise time (bpm X hrs)	4500 (4275, 4725)	4580 (4157, 5004)	0.66	4562 (4307, 4816)	4489 (4144, 4833)	0.68
Mean heart rate/distance (bpm /km)	3.9 (0.1, 7.8)	2.7 (0.3, 5.0)	0.49	1.4 (0.4, 2.3)	5.5 (2.0, 8.9)	**0.03**

All data are mean (95% CI).

Pearson correlations were performed to examine associations of baseline characteristics and exercise adherence/compliance in women with and without PCOS who completed the intervention, and changes in body composition, VO_2_ peak and indicators of insulin sensitivity from pre- to post-training. In women without PCOS, age was significantly positively correlated with changes in fasting insulin in response to training (r = 0.76; P = 0.047) while higher total body fat (r = -0.82; P = 0.03) and gynoid fat (r = -0.82; P = 0.03) at baseline were negatively correlated with changes in fasting glucose, and gynoid fat was also negatively correlated with change in gynoid fat (r = -0.77; P = 0.04). Baseline VO_2_ peak was negatively correlated with changes in android-gynoid fat ratio in women without PCOS (r = -0.97; P = 0.01). None of the baseline characteristics or exercise adherence/compliance indicators were associated with change in VO_2_ peak in women without PCOS (all P>0.05). Total number of exercise sessions was negatively correlated with change in fasting glucose (-0.76; P = 0.049).

**Fig 3:**
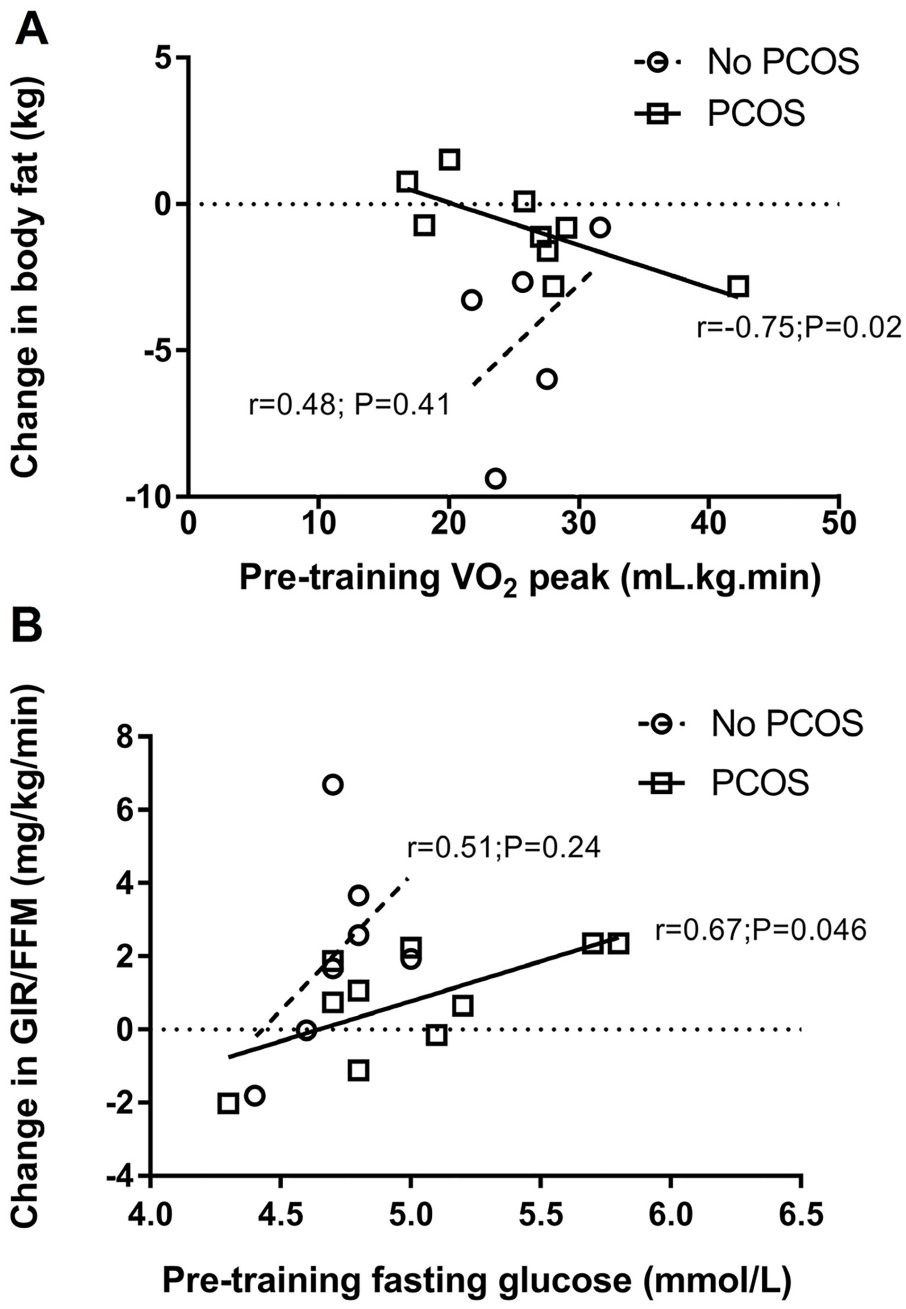
Pearson correlations for associations of baseline VO2 peak with change in body fat (A) and for baseline fasting glucose with change in GIR/FFM (B) according to PCOS status.

As demonstrated in Fig 3A, baseline VO_2_ peak was significantly negatively correlated with change in total body fat in women with PCOS, but not those without PCOS (two without PCOS had missing VO_2_ peak data at baseline). Baseline VO_2_ peak was also negatively correlated with change in gynoid fat (r = -0.69; P = 0.04) in women with PCOS. Baseline total body (r = -0.67; P = 0.048) and gynoid (r = -0.73; P = 0.03) fat were negatively correlated with change in android-gynoid ratio. Conversely, baseline android fat (r = 0.70; P = 0.04) and android-gynoid ratio (r = 0.72; P = 0.03) were positively correlated with change in gynoid fat. Baseline fasting glucose was negatively correlated with change in VO_2_ peak (r-0.76; P = 0.046) but also negatively correlated with change in glucose (r = -0.76; P = 0.02), and positively correlated with change in GIR/FFM (Fig 3B). The only association observed between exercise adherence and compliance indicators and exercise responsiveness in women with PCOS was a positive correlation between total exercise distance and change in VO_2_ peak (0.82; P = 0.044).

## Discussion

The primary findings of this secondary analysis of a 12-week intensified exercise intervention in overweight and obese women with and without PCOS was a substantial variation in insulin sensitivity, body fat and aerobic capacity changes in response to exercise, and although all participants improved VO_2_ peak (indicating improved fitness), approximately one–quarter did not improve insulin sensitivity and two-thirds did not reduce body fat by ≥5%. Although previous research has suggested that exercise non-responsiveness can be overcome by increasing exercise dose [19], we found few associations between indicators of exercise compliance and workload with study outcomes. Rather, high fasting glucose was associated with poorer improvements in VO_2_ peak, while lower baseline VO_2_ peak, higher relative central adiposity and lower insulin sensitivity at baseline were all associated with smaller fat losses. Nevertheless, despite smaller declines in total body fat, exercise non-responders significantly reduced relative central adiposity and improved aerobic capacity and insulin sensitivity. These findings suggest that overweight and obese women with and without PCOS can obtain health benefits from short-term exercise interventions even in the absence of significant body fat losses.

Women who failed to significantly reduce body fat in this study had significantly higher baseline android-gynoid ratios and lower GIR/FFM pre-training than exercise responders, and these associations are likely to be inter-related. In a recent study of normal weight PCOS women, intra-, but not subcutaneous, abdominal fat was positively associated with fasting insulin [20], supporting the concept that ectopic fat depots are more closely associated with cardiometabolic health than subcutaneous depots [21]. A systematic review has demonstrated that 15–20% of individuals with type 2 diabetes mellitus fail to improve metabolic health in response to supervised exercise programs [10], suggesting that dysregulation of glucose homeostasis (i.e high blood sugar levels, low insulin secretion and/or reduced insulin sensitivity) may be associated with poor exercise responsiveness in terms of body composition changes. Individuals at high risk of developing type 2 diabetes who fail to improve insulin sensitivity in response to an exercise program have increased transforming growth factor (TGF)-β activity in skeletal muscle, suggesting inflammation may contribute to poorer improvements on mitochondrial fuel oxidation and insulin sensitivity in this population [11]. Resistance to the beneficial effects of exercise has been shown to be highly heritable, with heritability estimates of almost 50% for VO_2_ peak in responses to exercise training [6]. However, the findings of the present study suggest adjunct therapies which improve insulin sensitivity and body composition, such as caloric restriction, may enhance metabolic exercise responses in overweight and obese women.

Despite losing small amounts of body fat following the 12-week exercise intervention, non-responders demonstrated significant decreases in android-gynoid ratio. This highlights that women who lose small amounts of fat in response to exercise may still undergo a shift to a more metabolically healthy body composition, and is supported by the finding that VO_2_ peak and GIR/FFM significantly improved in this group. An aerobic exercise and resistance training intervention in 31 overweight and obese postmenopausal women had similar numbers of non-responders (55%) as observed in our study (63%), despite its longer duration of 12 months [13]. Also similar to our study, the previous study observed higher BMI to be a predictor of poor responsiveness, although they did not assess differences in body fat deposition. Nevertheless, the investigators reported that primary outcomes including muscle strength and VO_2_ peak increased similarly for responders and non-responders [13]. These findings indicate that loss of body fat is not a limiting factor in improving aerobic capacity and insulin sensitivity in women in response to exercise, and highlight the need to focus on end-points other than body weight in interventions targeting overweight and obese individuals.

Several baseline characteristics were observed to be correlated with exercise-induced changes in body composition and indicators of insulin sensitivity in women with PCOS, yet these associations were somewhat controversial. For example, higher baseline total body and gynoid fat were associated with greater declines in android-gynoid ratio, but higher baseline android fat and android-gynoid ratio were associated with smaller declines in gynoid fat. These conflicting findings are consistent with the concept that not all individuals are similarly responsive to exercise training and potentially indicate that multi-component lifestyle modification programs may be most beneficial for women with PCOS. For example, a 16-week program of combined caloric restriction, behavioural modification, physical activity, and (for obese women) weight loss medication, resulted in weight loss of over 6% in women with PCOS [22], which is greater than the 2% weight loss observed in our intervention involving exercise only [4]. Nevertheless, our findings have demonstrated that insulin resistance and aerobic capacity can improve in response to exercise even when fat does not substantially decrease.

Higher baseline VO_2_ peak was associated with greater declines in total and gynoid fat in women with PCOS, which was somewhat surprising given improvements might be expected to be greater in those with the poorest cardiorespiratory fitness at baseline. Somewhat controversially, higher baseline fasting glucose was associated with smaller increases in VO_2_ peak but also with greater declines in fasting glucose, and greater increases in insulin sensitivity. The association of higher fasting glucose with smaller increases in VO_2_ peak may be reflective of the fact that higher levels of inflammatory markers and visceral fat are associated with lower VO_2_ peak in women with PCOS [23], but is unexpected given that insulin sensitivity appeared to be more likely to improve in women with PCOS who had higher fasting glucose at baseline. This may demonstrate that improvements in insulin sensitivity in response to exercise in women with PCOS do not necessarily associate with significant improvements in aerobic capacity.

Only the number of exercise sessions attended and average heart rate achieved were predictors for improvements in insulin sensitivity in women without PCOS. On the other hand, total distance covered during the intervention was associated with improvements in aerobic capacity in women with PCOS. A previous six-month exercise intervention also demonstrated improvements in aerobic capacity in women with PCOS who were most compliant with training [24]. Interestingly, while we observed that women with PCOS covered significantly more distance than those without PCOS in this intervention, the actual intensity or workload of exercise appeared to be significantly lower. Given that a previous study suggests that increasing exercise dose can abolish non-responsiveness [19], these findings highlight the need to maximise lifestyle program compliance and retention in women with PCOS [25], and suggest that during training, motivational strategies that ensure higher levels of exertion are likely to be beneficial.

A key limitation of this study was that there was substantial loss to follow-up resulting in small numbers for comparison of women with and without PCOS and responders and non-responders and therefore we were unable to control for potential confounders in multivariable analyses. There is also potential for bias in our results from this loss to follow-up although we did not identify any differences in characteristics of included and excluded participants. The sample size had only 79% power to detect a 10% difference in the primary outcome of insulin sensitivity between exercise responders and non-responders (based on body fat change) suggesting that it may have been inadequate to detect significant differences in other outcomes. However, we observed 90% adherence amongst those who completed the exercise intervention with no significant differences between women with and without PCOS [4], or between exercise responders and non-responders. Furthermore, participants who completed the intervention demonstrated 95% compliance to prescribed progressions in the exercise. We conducted a short-term exercise intervention and it is unclear whether interventions of longer duration would demonstrate similar associations between baseline characteristics and exercise non-responsiveness. Nevertheless, an analysis of six exercise interventions with a minimum duration of four months reported substantial proportions of metabolic non-responders in all studies, suggesting increasing the duration of an exercise intervention is not sufficient to overcome exercise non-responsiveness [7]. The strengths of our study include the gold-standard assessments for body composition and insulin resistance, as well as objective assessments of exercise adherence and compliance.

## Conclusions

Although a high proportion of overweight and obese women with PCOS had small reductions in total body fat following a 12-week exercise intervention, these exercise non-responders significantly reduced relative central adiposity and improved aerobic capacity and insulin sensitivity. Women with PCOS should be encouraged that participation in exercise, regardless of the effect on body composition, it can result in significant improvements in metabolic health.

## Supporting information

S1 File Study protocol. Description of the protocol for this study.
(PDF)

S2 File TREND checklist. Transparent Reporting of Evaluations with Nonrandomized Designs (TREND) Statement and Checklist for the current study.
(PDF)

S3 File Database. Pre- and post-training data for included study participants.
(XLSX)
