# Supplementary material for: Exploring factors related to changes in body composition, insulin sensitivity and aerobic capacity in response to a 12-week exercise intervention in overweight and obese women with and without polycystic ovary syndrome
Source: PLoS One. 2017 Aug 3;12(8):e0182412. doi: 10.1371/journal.pone.0182412 (PMC5542389; doi:10.1371/journal.pone.0182412)
Supplement: S1 File — Description of the protocol for this study. (PDF) [file pone.0182412.s001.pdf]

MODULE ONE:
CORE APPLICATION FORM AND CHECKLIST

State Government Victoria — Department of Human Services

BEFORE YOU BEGIN

This Application Form is for use by researchers proposing to conduct a research project involving humans. **All researchers must complete Module 1** and may have to complete other Modules (see checklist at Question 1.6).

Before you start this application, please read the **Module One: Core Application Guidelines** and the National Health & Medical Research Council's *National Statement on Ethical Conduct in Research Involving Humans* (1999).

Please do not delete the version date in the footer e.g. April 2004.

Office Use Only:

**HREC Ref. No.**______________		**Date of Approval:** / /	
**Approval Period:**	**From** / /	**To** / /	
**Approval signature:** ____			

SECTION A: PROJECT OVERVIEW

1.1 Application Date:

1.2 Full Project Title

Role of exercise in treatment of women with polycystic ovary syndrome: Mechanisms of action.
**FOR CLINICAL TRIALS ONLY:**<br>**Company/Sponsor Protocol Number (if applicable):**<br>**Version:**<br>**Date:**

1.3 Brief Lay Summary of the Project

Briefly describe the project. Refer to the Guidelines for the type of information and level of detail required in your response *(no more than one page)*

Approximately 7% of Australian women suffer from polycystic ovarian syndrome (PCOS). PCOS is characterised by insulin resistance, obesity, excess testosterone, irregular periods, infertility and, more recently, early signs of cardio-vascular disease (CVD). It is well known from studies in populations of type 2 diabetics, obese men and CVD patients that exercise training is a useful treatment and preventative intervention. As such, we wish to determine if exercise training can benefit PCOS patients and, specifically, the key mechanisms of exercise training that help improve insulin sensitivity, markers of CVD, sex hormone profiles and therefore menstrual and fertility problems.

As such the participants for the study will include women (aged 18-40) with and without PCOS. They will be recruited by advertisement and from clinics at Monash Medical Centre Clayton (gynaecological practices) and Dandenong Hospital (endocrinology clinics). The women without PCOS will be recruited from the local population, around the two hospitals and Monash University using advertisements in the local papers and announcements on the University global message system. Written consent will be obtained prior to any clinical screening to confirm the diagnosis of PCOS (as defined by the criteria suggested by the National Institute of Health; USA) required for their participation in the study. Part of this initial screen will include girth measures and a DEXA and CT scans for body compositionand simple non invasive vascular function assessed for indicators of CVD.

Following a three day standardised carbohydrate diet, the 2h insulin clamp and muscle and overlying fat biopsies will be performed. Muscle and overlying fat biopsies will be taken from the thigh muscle (under local anaesthesia) prior to and immediately after completion of the clamp, allowing the determination of the effects of insulin on skeletal muscle including gene and protein expression. Finally, the women will report to the Exercise Physiology Laboratory (Monash University) to perform a maximal incremental test to volitional exhaustion on a treadmill to determine aerobic exercise fitness.

Within a week of completing the initial tests, the women will perform a 12 week prescribed exercise program requiring their participation of exercise 3 days per week for 1 h each visit. This group of PCOS patients (30) and matched controls (30) will perform an endurance (aerobic style) exercise utilizing treadmills for walking/running activities. The participants will have all training sessions supervised by the chief investigator and/or an assistant (personal trainer) to facilitate exercise programme adherence. Participants will also provide a weekly urine sample, to be collected first thing in the morning on the same day of every week (frozen in their own freezers), training logs, and the days of menstrual bleeding in a diary. This will allow physical activity and hormonal profiles to be monitored during the exercise interventions. The exercise tests, body composition measures and scans, vascular function measures and the insulin clamp including muscle and overlying fat biopsies will be repeated immediately after (within 3 days) of completing the 12 wks of exercise training.

The expected outcomes from this study are:
1) Improvements in insulin sensitivity and the response of skeletal muscle to insulin stimulation (as determined by changes in gene and protein expression and protein activation of insulin responsive proteins). 2) Improvements in reproductive hormone profiles, as determined by urinary hormonal profiles, and/or return of normal menstruation. 3) Improvements in markers of early CVD. 4) Weight loss and fat mass changes in the abdominal region. 5) Improved muscle strength and/or endurance capacity which is hoped to improve daily living.

1.4 Relationship to Other Projects

Indicate whether the project is

☒ a new stand-alone project

☐ a sub-component of a previously approved project

☐ related to other previously approved projects (e.g. a follow-up study)

If the project is a sub-component of, or in some other way related to, a previously approved project, provide project numbers for the other project(s). Also indicate which HREC(s) approved the other project(s).

1.5 Broad Category of Research

Tick the category which best fits the application:

☐ Social Science	☒ Clinical Research
☐ Psychological	☐ Clinical Drug or Device Trial ⇨ CTN ☐ or CTX ☐
☒ Public Health	☐ Other (please specify) …………………………

1.6 Project Summary

Does the project involve

- Participants? Yes ☒ No ☐

  If yes, please complete section D of Module 1

- Collection, use or disclosure of information? Yes ☒ No ☐

  If yes, please complete section E of Module 1

- Drug or device trial? Yes ☐ No ☒

  If yes, please complete Module 2

- Use of human tissues? Yes ☒ No ☐

  If yes, please complete Module 3

- Human genetic research? Yes ☒ No ☐

  If yes, please complete Module 4

- Use of radiation? Yes ☒ No ☐

  If yes, please complete Module 5

1.7 Multi-Site Projects

Is the project a multi-site project? That is, does the project involve recruitment of participants at more than one site and/or collection of information from more than one organisation?

Yes ☒ No ☐

If *Yes*, does the project have to be reviewed by other HRECs?

Yes ☒ No ☐

Name **all Australian HRECs** to which this project has been or will be submitted. For each HREC, list all Australian sites involved in this project that are covered by the application to that HREC. If the number of sites for a particular HREC is very large (or unknown), such that listing individual sites is not feasible, indicate the number of sites covered by that HREC (*e.g. 50 primary schools or 20 out of 60 child care centres, etc*). Indicate the status of the application to other HRECs.

**HREC**	**Site**	**Status of application** (*e.g. not yet applied/approved/ rejected/pending*)
Monash University SCERH	Clayton	Co-ordinated Review

SECTION B: RESEARCHERS AND CONTACT INFORMATION

1.8 List all researchers involved in this project

Copy this table and repeat for each ***Principal Researcher****.*

Title and Name	Dr Nigel K. Stepto
Appointment	Lecture/Researcher
Department	Physiology
Institution	Monash University
Mailing address	Building 13F, Monash University, Clayton, Victoria 3800
Describe what this researcher will do in the context of this project	This researcher is the primary investigator and will be responsible for the co-ordination of the project. In addition he will be responsible for the exercise testing, and exercise training prescription. He will also be conducting/supervising all analytical techniques on the human blood and tissue.
Include a brief summary of relevant experience for this	Dr Stepto has a PhD in exercise metabolism and has worked as personal trainer at Monash University Fitness gym. This qualifies him to conduct exercise testing and prescription. His

project	experience with exercise metabolisms also provides him with all required experience to conduct the analytical techniques used to analyse all the human tissue sampled.
Phone	9905 2543
Fax	9905 2547
Mobile/pager	0409338696
email	nigel.stepto@med.monash.edu.au

Copy this table and repeat for each ***Associate Researcher****.*

Title and Name	A/Prof Ben Canny
Appointment	Associate Professor/Associate Dean
Department	Physiology
Institution	Monash University
Mailing address	Building 13F, Monash University, Clayton, Victoria 3800
Describe what this researcher will do in the context of this project	Intellectual input with design and analysis of project. Patient screening, and perform muscle biopsies. Manuscript preparation.
Include a brief summary of relevant experience for this project	Assoc Prof Canny is a medical practitioner with over ten year's experience with human exercise physiology experiments. He has performed over 500 exercise-related muscle biopsy procedures. He has published extensively in the field of exercise and endocrinology with over 60 peer-reviewed publications.
Phone	9905 2567
Fax	9905 2547
Mobile/pager	0412 111 447
Email	ben.canny@med.monash.edu.au
Title and Name	Dr Helena Teede
Appointment	NH&MRC CDA Fellow; Head Endocrinology Diabetes
Department	Department of Vascular Sciences and Medicine
Institution	Dandenong Hospital
Mailing address	128 Cleeland Street, Dandenong 3175
Describe what this researcher will do in the context of this project	Intellectual input with design and analysis of project. Patient screening, supervision of clamp studies, completion/ supervision of vascular functional studies, manuscript preparation.
Include a brief	Extensive experience in PCOS clinically and in research

summary of relevant experience for this project	setting. Extensive clinical trial experience, (40 peer reviewed publications) and experience / expertise in assessment of insulin resistance and non invasive assessment of vascular function.
Phone	9554 8024
Fax	9554-8027
Mobile/pager	0407 005 737
email	Helena.teede@southernhealth.org.au
Title and Name	Dr Beverley Vollenhoven
Appointment	Senior Lecturer
Department	Obstetrics and Gynaecology
Institution	Monash University
Mailing address	Monash Medical Centre, 246 Clayton Rd, Clayton, Victoria 3168
Describe what this researcher will do in the context of this project	Intellectual input with design and analysis of project. Patient screening and manuscript preparation.
Include a brief summary of relevant experience for this project	Reproductive endocrinologist who has experience with patients who have PCOS. Has run PCOS studies in the past.
Phone	9544 6688
Fax	9544 4707
Mobile/pager	0414 772 962
email	beverley.vollenhoven@med.monash.edu.au
Title and Name	A/Prof Boyd Strauss
Appointment	Director of Clinical Nutrition & Metabolism; Physician in Charge, Body Composition Laboratory; Physician in General Medicine.
Department	Department of Medicine
Institution	Monash University
Mailing address	Monash Medical Centre, 246 Clayton Rd, Clayton, Victoria 3168
Describe what this researcher will do in the context of this project	Provision of access to the DEXA and CT scanners at Monash Medical Centre. Supervision of the analysis of all scans, including DEXA, and CT scans to provide data on changes in tissue distribution.
Include a brief	Extensive experience with body composition measures in the

summary of relevant experience for this project	clinical trials. Research interests and current activities using DEXA and CT techniques include, Measurement of body composition in individuals and populations in health and disease. In addition, has published 64 peer reviewed papers which relate to body composition assessment and validation.

*Copy this table and repeat for each **Principal Researcher**.*

Title and Name	Dr Samantha K Hutchison
Appointment	Endocrine Fellow/PhD student
Department	Monash Institute of Health Services Research
Institution	Monash University
Mailing address	MIHSR, Locked bag 29, Monash Medical Centre Clayton, Victoria 3168
Describe what this researcher will do in the context of this project	This researcher is the trial co-ordinator and responsible for the co-ordination of the project. In addition she will be trained to conduct insulin clamps and muscle biopsies as well as clinical assessments of the patients. She will be assisting in analytical techniques on the human blood and tissue.
Include a brief summary of relevant experience for this project	Dr Hutchison is an Endocrine advanced trainee in her final year of training and has commenced her PhD in 2006. She has experience with the clinical assessment of PCOS during her medical training.
Phone	9594 7510
Fax	9594 7550
Mobile/pager	0411836010
email	Samantha.hutchison@med.monash.edu.au

*Copy this table and repeat for each **Principal Researcher**.*

Title and Name	Cheryce Harrison
Appointment	Honours Student
Department	Department of Physiology
Institution	Monash University
Mailing address	Building 13F, Monash University, Clayton, Victoria 3800
Describe what this researcher will do in the context of this project	This researcher is responsible for the co-ordination of the exercise training of the participants. In addition she will be provide assistance with the insulin clamps She will be assisting in analytical techniques on the human blood and tissue.
Include a brief summary of relevant experience for this	Ms Harrison is an honours student with experience in exercise physiology and has all the relevant experience in conducting exercise tests and training for the participants.

project	She will be closely supervised by Dr Stepto
Phone	9905 2543
Fax	9905 2547
Mobile/pager	0423001543
email	cheryce.harrison@med.monash.edu.au

*Copy this table and repeat for each **Associate Researcher**.*

Title and Name	Dr Lisa Moran
Appointment	Post-doctoral researcher
Department	MIHSR
Institution	Monash University
Mailing address	MIHSR, Locked bag 29, Monash Medical Centre Clayton, Victoria 3168
Describe what this researcher will do in the context of this project	Lisa will assist in conducting vascular functional studies. In addition she will be trained to assist in conducting insulin clamps.
Include a brief summary of relevant experience for this project	Lisa has completed a PhD in PCOS and has experience in clinical research with PCOS patients including taking of blood samples.
Phone	9594 7562
Fax	9594 7554
Mobile/pager	0401963713
email	Lisa.moran@med.monash.edu.au

Title and Name	Dr Dominic Rachon
Appointment	Physician, Post-doctoral researcher
Department	MIHSR
Institution	Monash University
Mailing address	MIHSR, Locked bag 29, Monash Medical Centre Clayton, Victoria 3168

Describe what this researcher will do in the context of this project	Dominic will be trained to assist in conducting insulin clamps and will insert intravenous cannulas including taking of blood samples.
Include a brief summary of relevant experience for this project	Dominic is a physician and has experience with cannulation and he has also has completed a PhD. He has experience in clinical research.
Phone	9594 7562
Fax	9594 7554
Mobile/pager	
email	

1.9 Training

Will any of the researchers require extra training to enable their participation in this project?

Yes ☒ No ☐

If *Yes*, list the researchers, describe the training that is required and who will provide this training.

**Researcher**	**Training required**	**Who will provide training?**
Dr Samantha Hutchison	Muscle biopsy technique	Dr Ben Canny
Ms Cheryce Harrison	Exercise testing and training	Dr Nigel Stepto
Dr Lisa Moran<br>Dr Dominic Rachon	Insulin clamp technique	Dr Nigel Stepto/Dr Samantha Hutchison

1.10 Person to whom the HREC may also direct correspondence:

Title and Name	Dr Andrea Lines
Appointment	Human Ethics Officer
Department	Research Grants and Ethics Branch
Institution	Monash University
Mailing address	Wellington Rd Clayton 3800
Phone	9905 2052
Fax	
Mobile/pager	

email	andrea.lines@adm.monash.edu.au

SECTION C: PROJECT DETAILS

1.11 Anticipated duration of project: 36 months

1.12 Anticipated commencement date at this site: 15/03/2005

1.13 Anticipated completion date at this site: 31/12/2007

1.14 Detailed Project Proposal

If the project is a clinical drug or device trial DO NOT complete question 1.14, but move directly to question 1.15. The detailed project proposal for clinical drug or device trials is completed in Module 2.

(a) Project Checklist

Major Proposal Components	Page and/or section number in the proposal	Not Applicable
Literature review	Included Below	☐
Rationale for project	Included Below	☐
Hypothesis/research questions	Included Below	☐
Aims	Included Below	☐
Methodology	Included Below	☐
Inclusion/exclusion criteria	Included Below	☐
Randomisation procedures		☒
Statistical or other analyses	Included Below	☐

(b) Project Proposal

Every application must be accompanied by a detailed proposal. You may type (or "paste") your detailed proposal directly into the text box below and/or you may attach pre-printed document(s) immediately following this page. Attachments should include brochures/pamphlets, questionnaires or surveys and any other relevant documents. **Ensure that all attachments are page numbered throughout.**

You should consult the Guidelines about the type of information that should be included in the detailed proposal.

Background & Rationale

Polycystic ovary syndrome (PCOS) is now recognized to be a complex syndrome that not only affects the reproductive system but whole body metabolism. Women with PCOS experience increased androgen production and, as a result, cosmetic problems, anovulation and infertility. In addition, there are complications of metabolic syndrome such as obesity, insulin insensitivity and a 6 fold increased risk of diabetes mellitus, high- cholesterol, abnormal lipid profiles and hypertension (*1*). There may also a risk of carcinoma of the endometrium in later life. Obesity alone in women also is associated with elevated androgens and insulin resistance,

menstrual dysfunction and infertility. In addition, the Western World's growing trend towards obesity, which women suffering with PCOS are highly susceptible to, may place women at a greater risk of type 2 diabetes mellitus and/or cardiovascular disease (*2*). The prevalence of PCO in women of reproductive age has been suggested to as high as 25% of the population depending on the criteria used for diagnosis (reviewed in(*1*)) and as many as 5-10% of women have the clinical syndrome (*3, 4*). So, compounding the problems of PCOS, is that it now appears that both PCOS, *per se*, and the obesity to which they are susceptible, have a synergistic effect on the degree and severity of insulin resistance and subsequent hyperinsulnaemia (*5*) and therefore reproductive and metabolic outcomes.

Current medical treatment regimens for PCOS generally advocate the use of the exogenous hormones normally in the form of oral contraceptive pill. These usually assist with menstrual abnormalities and excess androgen production, but are inappropriate for women trying to conceive and have been ineffective at dealing with the metabolic abnormalities, they can also cause weight gain and may exacerbate the risk of cardiovascular disease. Lifestyle interventions are first line in therapy for PCOS and lifestyle modifications through diet and exercise have proved to be effective in improving ovulation and pregnancy rates in PCOS patients (*1, 6*). The studies conducted to date have focused on diet, and specifically, the researchers have used caloric restriction in combination with group or individual exercise programs (*7-12*). The carbohydrate, fat and protein composition of the diet used for the caloric restriction appeared to be irrelevant, but in combination with exercise programs a over 3 to 6 month period the program was effective in the normalization of resting insulin concentration and steroidal hormone profiles, resulting in the return of ovulation (*7-12*). The overall improvements in cardiovascular and reproductive parameters were associated with weight loss and more specifically the reduction in central adiposity (*5, 10*) suggesting obesity as a primary factor for these metabolic and reproductive perturbations in PCOS.

Furthermore, 60% of PCOS patients studied in the literature (*10, 13*) seemed to respond to the lifestyle modifications, with the return of ovulation and an improved insulin sensitivity upon attaining a 9-10% weight loss. It was interesting that the remaining subjects failed to respond, but this may in fact be due to a need for a greater change in absolute fat
-mass rather than the prescribed 10% of body mass.

With the realization that PCOS may be linked to hyperinsulinaemia and dysfunctional insulin signalling (*14*), PCOS patients have also been treated with insulin sensitizing drugs such as metformin (*15-17*) and rosiglitazone (*18*). These drugs have had mixed results (*19*) but were successful in improving the metabolic syndrome as well as decreasing, in some cases, the androgen concentrations and improving ovulation and pregnancy rates (*15, 17*). More recently, and somewhat counter intuitively, Maciel *et al.* (*16*) found that metformin was more effective in non-obese PCOS patients than their obese counterparts. While it would appear to be an effective therapy the safety of insulin sensitizing drugs for long term use in young women or during pregnancy still need to be validated and there is consensus agreement that medical therapy should still be reserved for second line therapy if lifestyle intervention is inadequate (*20*).

In addition, women with PCOS have an adverse CV risk profile related to insulin resistance, an independently associated CV risk factor (*1*). The insulin resistance and metabolic syndrome observed in PCOS would be expected to culminate in a higher risk of CVD in these women, however the data in this area is poor and inconclusive (*1*). Epidemiological data on PCOS and CVD is limited however a study of medical records in women with prior PCOS on histology did not note an increase in CVD (*21*). These studies have significant flaws and are hampered by long lag times between diagnosis of the PCOS and the onset of CVD, along with inconsistent diagnostic criteria for PCOS. However, recent data from AR2 laboratory has shown that PCOS patients have an increase in the early markers for CVD, as determined by Pulse wave velocity; Flow mediated vasodilation and carotid intima media thickness. These accurate reproducible markers of early CVD predict clinical outcomes in hypertensive and renal populations These surrogate measures have been shown to improve after exercise training in populations of type 2 diabetics and obese men (*22, 23*). Further clarification of the cardiovascular risk and the role of exercise training in these women are needed.

It is now apparent that modern society is more accepting of the pharmacological treatment of PCOS. While this method is easy to administer it does have a number of draw backs including the cost of the medication and possible side-effects of the drugs. The alternative is the prescription of an exercise programme, which we know helps restore fertility and insulin sensitivity to these women. However, a further complication of such an approach is the low adherence of patients to exercise regimens. It is therefore important to research the effects of exercise in patients with PCOS, such that more achievable forms of exercise are available, and that one may gain strong evidence to advocate exercise prescription as an effective and lifelong therapeutic approach to syndromes like PCOS. Furthermore, by providing a greater understanding of the mechanisms of action of exercise may lead to a greater acceptance of its use by both the clinicians and patients.

Specific aims:

The primary aim of this study is to investigate the use of increased physical activity including endurance exercise as a treatment/management tool for women with PCOS. The specific aims would be to:

1) Determine the specific improvements in insulin resistance and subsequently other reproductive and metabolic features of PCOS for women prescribed endurance training.

2) Investigate the possible mechanisms of action for improved skeletal muscle and fat metabolism through which exercise training elicits its beneficial effects, by examining changes in muscle insulin signalling after 12 weeks of endurance training.

3) Determine the effects of endurance exercise training on the measures of early CVD (namely; Pulse wave velocity; Flow mediated vasodilation and carotid intima media thickness) in women with PCOS.

4) Investigate the palatability of an exercise regime in these women.

Hypothesis:

The main hypothesis is that the prescription of an exercise training program will

improve clinical, endocrine and metabolic features of PCOS in women suffering with this syndrome. The secondary hypothesis is that the mechanism of action of exercise on influencing the pathophysiology of PCOS is via the improvements in insulin resistance and muscle insulin signalling and will lead to concurrent improvements in surrogate markers of early CVD.

Methodology:

Subjects:

Women (aged 18-40 yrs, body mass index [BMI] between 27 and 35 $kg.m^{-2}$) with and without PCOS will be recruited from advertisement and from clinics at Monash Medical Centre and Dandenong Hospital.

Participants will be screened by telephone by the coordinating researcher. If eligible, participants will be invited to attend for screening with completion of a detailed medical history and physical examination (Dr S Hutchison, Dr B. Vollenhoven or Dr Helena Teede). All participants will receive dietary and lifestyle advice (standard advice provided by an experienced clinician (SH, HT and BV) and accompanied by written information from the National Heart Foundation).

Thirty women with PCOS and thirty controls will be recruited. All women in each group will complete baseline testing. and perform endurance.training. The controls and PCOS women will be matched for BMI. The women without PCOS will act as controls. All participants will be screened by an experienced clinician (SH, BV and HT) for the diagnosis of PCOS (National Institute of Health [USA] criteria) to exclude other causes of hyperandrogenism and other causes of an- or oligo-ovulation (See exclusion criteria). Prior to the study, control and PCOS patients will be required to have not taken medications for the condition such as combined oral contraceptives and/or insulin sensitising drugs (3 months prior) for inclusion into the study and will be asked to use barrier contraception. During this screening these subjects will have a cholesterol and fasting blood glucose levels to exclude diabetes and history and examination will exclude CVD. They will also be required to take a pregnancy test if child bearing is a possibility. There will be a 3 month lead in to the study so as to allow for the cessation of all medications for wash out of the drugs and the implementation of healthy eating practices. It will also allow the participants to undergo all initial screening and testing.

Initial testing:

Participants will have a number of preliminary tests done, including 1 DEXA and 2 CT (1 abdominal and 1 mid-thigh) scans of body composition for determination of fat mass and distribution (*24-27*) of the controls and PCOS patients. All women will have a history of their menstrual cycles checked, via a recall questionnaire. They will also have bloods taken to determine their blood lipid and hormonal profiles (including TSH and Prolactin, FSH, LH, DHEAS, Androstenedione, Testosterone, SHBG and FAI). They will also undergo non-invasive vascular assessment including Pulse Wave Velocity and Flow mediated vasodilation (endothelial function), this takes 25 minutes including rest time. Participants are tested after a resting for 15minutes in a temperature controlled dark room.

After a 72 hr standardised carbohydrate diet and an over night fast, participants will be weighed, and have basic body measures taken,

On a separate day the participants will undergo a 2 hour hyperinsulinaemic

euglycaemic clamp (the gold standard test for insulin sensitivity), which involves the infusion of insulin to induce a supraphysiological insulin profile, with a glucose infusion which is continuously adjusted to maintain blood glucose concentrations at ~5mmol.L^{-1}. During this clamp serial blood samples will be taken at regular intervals to monitor blood glucose and insulin concentrations for the determination of insulin sensitivity and glucose tolerance (conducted in research labs at Monash University and supervised by Samantha Hutchison, Lisa Moran, Dominic Rachon). Muscle and overlying fat biopsies (performed by A/Prof B Canny and Dr Samantha Hutchison) will also be taken before and 30 minutes into the hyperinsulinaemic clamp for the determination of muscle insulin signalling. This procedure will be conducted in the follicular phase in the control subjects.

The women will go on to perform an incremental test to exhaustion on a treadmill, to determine their maximal oxygen uptake (VO_{2max}; endurance capacity) to allow the exercise intensity to be set for endurance exercise training.
Within a week of the completion of the exercise testing the participants will perform a 12-week prescribed training programme of endurance exercise in the Monash University Physiology Department . All subjects will be provided with a heart-rate monitor for monitoring exercise intensity. The exercise programme will require the participants to attend the gym for a minimum of 1 hr a day for 3 days of the week and will be supervised at all sessions (Dr N Stepto, Cheryce Harrison). The endurance training group will exercise on the by walking on a treadmill such that they complete 60 min of exercise. The exercise intensity for endurance exercise will be set using heart rates such that they will be expected to have maintained a heart-rate of 70-80% of their pre-determined maximal heart-rate. Endurance training regimens will use the principles of training such that the participants exercise intensity and/or duration will be increased over the training period. During training a monthly review will be included to adjust the exercise programme in accordance with their training gains, and facilitating increases in training load of no more than 10% per week. The endurance exercise intensities will be monitored and set from maximal exercise tests conducted on the treadmill.

During these 12 weeks of training, all subjects will be monitored with weekly urine samples, training logs and menstruation diaries and a two week food diary. After the 12 weeks of training the insulin clamp, muscle and overlying fat biopsies, vascular function, body composition measures and exercise tests will be repeated in the follicular phase of menstrual cycle (if menstruation returns), to monitor changes in hormonal profiles, menstruation, insulin sensitivity, muscle metabolism and signalling in these women.

Inclusion and Exclusion Criteria:
Women with PCOS:
Inclusion: Women aged 18-40 years with PCOS with BMI >27 and <130kg (limitation of CT/DEXA scan). Diagnostic criteria for PCOS will be strict and will be based on NIH criteria including a history of perimenarchal onset of oligomenorrhea (<8 cycles per year or cycle length <21 or >35 days), insulin resistance and either clinical manifestations of hyperandrogenism (acne or hirsutism or both) and/or elevation of at least one biochemical marker of androgenicity. They must also be off all hormonal and insulin controlling drugs for at least 3 months prior

to the study, and be non-smokers.
Exclusion: Cardiovascular disease, type 2 diabetes, pregnancy and smokers.

Women without PCOS:
Inclusions: BMI matched premenopausal females aged 18-40 years without PCOS. They must also be off all hormonal treatments for at least 3 months prior to the study.
Exclusion: Cardiovascular disease, type 2 diabetes, pregnancy and smokers.

Analytical Techniques:
Exercise Tests and Training:
Incremental test to exhaustion.
This exercise test will be conducted in the Monash University Exercise Physiology laboratory. This test involves the participants to walk/jog on a motorized treadmill, which can have both gradient and speed of the exercise adjusted to increase the intensity of exercise. This test will use the Bruce Protocol which is a continuous incremental protocol, starting at 1.6 km.h^{-1} and 0% grade which will be maintained for 120s after which the speed and grade will be increased every 180s (see table 1 below) until a volitional fatigue or a speed of 10.4 km.h-1 and 24% grade is reached and maintained for the 180s, after which the subject has a 2 min cool down. During the test heart rate will be measured using a Polar® A3 heart rate monitor (Polar-Electro OY, Kempele; Finland), expired gas will be collected using an automated gas analysis system (MOXUS modular VO_2 system; AEI technologies, Pittsburgh, Pennsylvania, USA) to determine the oxygen consumption and carbon dioxide production on breath-by-breath basis.

Table 1: Treadmill speeds and grades in the Bruce Protocol used for the determination of maximal oxygen consumption (VO_{2max})

Exercise	Speed (km.h^{-1})	Grade/slope (%)	Time (s)
Warm-up	1.6	0	120
Test	2.7	10	180
	4.0	12	180
	5.5	14	180
	6.7	16	180
	8.0	18	180
	8.8	20	180
	9.6	22	180
	10.4	24	180
Cool down	2.5	0	60
	1.6	0	60

Maximal or peak oxygen consumption (VO_{2max} or VO_{2peak}) is defined as the highest oxygen uptake attained during two consecutive 30s sampling periods. The results of this test will be used to define training intensities which are usually 70-90% of the maximal oxygen consumption (see training protocols). This test is terminated using the following criteria in order of importance:

1) Patient is physically distressed and voluntarily stops
2) The patient's heart rate reaches 180 beats per minute
3) The patient wishes to continue but is visibly distressed

4) The respiratory exchange ratio (RER) is greater than 1.1
5) VO_2 does not increase further with increase exercise intensity

This exercise test is repeated after 4 and 8 weeks of training to monitor changes that result from the training regimens.

Screening:
Menstrual diaries and recall
Completion of a diary (attached), initially the diary will be on recall for the 6-12 months prior to the study, and the second one will be completed from the beginning of the study including the 3 month lead in period.
Food diary
*Completion of a two week food diary to be used as a guide f*or the duration of the study*Gynaecological and endocrinological screening*
Screening will be conducted for the diagnosis of PCOS and the exclusion of other causes of hyperandrogenism (NIH criteria; see inclusion/exclusion criteria) in the PCOS group, while the controls will undergo the same screening for exclusion of PCOS. This will include a blood test for pregnancy, if this is a possibility.

DEXA and CT scan
Women will be scanned using a Dual Energy X-ray Absorptiometry (DEXA) scan for the assessment of whole body composition and will be repeated after the study. The scans will be conducted at Monash Medical Centre within the Department of Medicine. The DEXA scan will take approximately 30min and expose the subject to a minimal dose of ionising radiation (0.1 mSv per scan). After each DEXA scan the women will also undergo 2 CT scans, one of the L4-L5 region of the abdomen (0.3 mSv per scan) and the mid-thigh (0.1 mSv per scan) for assessment of the fat distribution in these body areas, which an MRI scan could not provide (*24-26*). The total ionising radiation that the volunteers will receive over the entire study is 1.5 mSv, which is well below the annual exposure limits of 5 mSv per annum and is equivalent to 274 days of natural background radiation exposure living in Melbourne.

Cardiovascular measures:
These non-invasive measures are completed with-out exposure to radiation using pressure tonometry techniques as well as ultrasound.

Pulse wave velocity: PWV will be determined from recorded pressure waveforms over both the aorto-femoral (A-F) and the femoro-dorsalis pedis (F-D) arterial segments. Pulse transit time will be defined as the time between the foot of simultaneously recorded pressure waves (end of diastole and the beginning of systole), averaged over 10 cardiac cycles. Velocity will be derived from pulse transit times divided by measured distances between the two recording sites.
Brachial artery flow mediated vasodilation: Brachial artery diameter will be measured from B-mode ultrasound images captured on a Diasonics DRF-400 machine using a 10-MHz transducer, whilst an ECG trace was simultaneously recorded. Longitudinal scanning identified the clearest image of the brachial artery above the elbow, with continuous scanning held for 30 seconds prior and 4 minutes after ischemia, induced via a pneumatic tourniquet inflated around the upper arm to

40mmHg above systolic pressure for 4 minutes. Vessel diameter will be measured during systole and diastole and averaged over 5 cardiac cycles. FMD will be determined as the percentage change from baseline to 60 seconds post ischemia, the point of maximal dilation.

Muscle and overlying fat Biopsy procedure:
The biopsies will be undertaken by removing 100 to 150 mg of muscle and overlying fat from the thigh muscle under local anaesthesia, using a biopsy needle. For local anaesthesia approximately 1-2ml of xylocaine will be injected subcutaneously to anaesthetise the skin and underlying tissue. A small cut (approximately 7mm) will then be made and the biopsy cannula inserted into the muscle. The time taken for muscle and overlying fat removal is 4-6 seconds. At the time of initial biopsy a second incision will be made for the additional muscle samples that will be obtained 30 min after commencement of the insulin clamp.

Euglycaemic Hyperinsulineamic Clamp:
An anticubital vein (forearm) will be cannulated for the infusion of insulin and glucose, and a hand vein on the contra-lateral arm will be retrogradely cannulated and warmed for serial arterialized venous blood sampling. The cannula used for blood sampling will be kept patent using regular flushing of 0.9% sterile saline (~2ml). Insulin will be continuously infused (40 $mU.m^{-2}.min^{-1}$) for 120 min immediately following a primed bolus of insulin (9 $mU.kg^{-1}$ lean body mass). A variable glucose infusion (25% sterile solution) will be used to maintain a constant blood glucose concentration (euglycaemia; 5 $mmol.L^{-1}$). This will be done by monitoring blood samples taken every 5 min during the clamp. There is a risk of potassium depletion during this clamp, so to prevent this 30mmol KCl (Slow-K) will be administered orally at the onset of this procedure.

Blood and Urine sample analysis:
Most analysis blood will be done using commercially available kits or automated analysers. As such blood glucose (especially during the clamp) will be analysed immediately using the YSI automated glucose/lactate analyzer. Insulin concentrations and steroid hormone and lipid profiles will be conducted by Southern Health Pathology services. The weekly urine samples, collected in first thing in the morning on the same day of each week, by the subjects to freeze in their own freezers, will be analysed at Monash University for menstrual hormonal profiles using commercially available testing kits.

Muscle and fat tissue analysis:
Protein extraction and analysis: A fraction of the fat and muscle biopsy will be used for protein extraction using standard laboratory techniques. The extracted proteins will be used for a number of enzyme activity and immunoblot (Western Blots) assays.
mRNA extraction and analysis: Muscle messenger RNA (mRNA) will be extract using a standard extraction procedures, some RNA will be amplified for gene-expression micro-array analysis for mRNA expression profiling. The remaining RNA will be used to examine changes in the expression of specific genes in the skeletal muscle using real-time RT-PCR.

Statistical analysis:

Sample size: 1) Based on limited current literature, Corbould et al (AI-AC) (30) have characterised differences in skeletal muscle insulin signalling in a cross-sectional setting (20 PCOS women and 15 controls) demonstrating significant differences (α=0.05), between the groups, with a power of 75%. With 25 participants in each group (α =0.05), allowing for a 20% dropout rate (during the run-in phase), we will be powered to detect significant difference in these parameters with a power of 86%. 2) Huber-Burchholz et al (16) have conducted a lifestyle intervention (diet and unquantified exercise) study in a similar population of women with 6 PCOS and 7 controls, demonstrating that there was a significant change in our selected main outcome variable (glucose infusion rate: a marker of insulin resistance from euglycaemic hyperinsulinaemic clamps). α was 0.05 and within group changes of 20% for controls and 40% for PCOS was noted. The 20% between group difference in change provided a power of 50%. Here, to detect the same between group difference (5 $\mu mol.kg^{-1}.min.^{-1}$ or 1 SD) (16) with an α of 0.05, 15 subjects in each group will have a power of 93%. If a 20% drop-out occurs, the study will have a n 84% power with 12 per group.

Data analysis: In the baseline and interventional phase of the study the primary endpoints are insulin resistance and insulin signalling, and how it differs and/or changes after 12 weeks of intensified exercise training in PCOS and control women. Secondary end-points are the cardiovascular measures and body composition measures. In the PCOS group, clinical end-points will also be studied focusing on with-in group changes (ovulation and hormone profiles). All data will be collected serially over the experimental trials or during the 12 weeks of training (fitness assessments, urinary samples, insulin signalling pathway activation and gene expression data (QRT-PCR mRNA) and will be analysed using repeated measures ANOVAS or paired t-tests.

References:

1. R. J. Norman, R. Wu, M. T. Stankiewicz, *Medical Journal of Australia* **180**, 132 (2004).
2. R. J. Norman, L. Masters, C. R. Milner, J. X. Wang, M. J. Davies, *Human Reproduction* **16**, 1995 (2001).
3. Z. E. C. Hopkinson, N. Sattar, R. Fleming, I. A. Greer, *Bristish Medical Journal* **317**, 329 (August 1, 1998, 1998).
4. R. Azziz *et al.*, *Journal of Clinical Endocrinology and Metabolism* **89**, 2745 (June 1, 2004, 2004).
5. A. Balen, M. Rajkowha, *Best Pract Res Clin Obstet Gynaecol* **17**, 263 (Apr, 2003).
6. R. J. Norman, M. J. Davies, J. M. Lord, L. J. Moran, *Trends in Endocrinology and Metabolism* **13**, 235 (2002).
7. K. Stamets *et al.*, *Fertil Steril* **81**, 630 (Mar, 2004).
8. L. J. Moran, M. Noakes, P. M. Clifton, L. Tomlinson, R. J. Norman, *Journal of Clinical Endocrinology and Metabolism* **88**, 812 (2003).
9. S. E. Kasim-Karakas *et al.*, *Journal of Clinical Endocrinology and Metabolism* **89**, 615 (Feb, 2004).

10. M.-M. Huber-Burchholz, D. P. G. Carey, R. J. Norman, *Journal of Clinical Endocrinology and Metabolism* **84**, 1470 (1999).
11. A. M. Clark *et al.*, *Human Reproduction* **10**, 2705 (1995).
12. A. M. Clark, B. Thornley, L. Tomlinson, C. Galletly, *Human Reproduction* **13**, 1502 (1998).
13. E. W. C. van Dam *et al.*, *American Journal of Physiology, Endocrinology and Metabolism* **286**, E615 (2004).
14. A. Dunaif, J. Xia, C. B. Book, E. Schenker, Z. Tang, *J Clin Invest* **96**, 801 (Aug, 1995).
15. R. J. Norman, W. J. Kidson, R. C. Cuneo, M. R. Zacharin, *Medical Journal of Australia* **174**, 580 (2001).
16. G. A. Maciel *et al.*, *Fertil Steril* **81**, 355 (2004).
17. J. M. Lord, I. H. K. Flight, R. J. Norman, *Bristish Medical Journal* **327**, 951 (2003).
18. S. H. Belli *et al.*, *Fertil Steril* **81**, 624 (2004).
19. E. Diamanti-Kandarakis, H. A. Kandarakis, *Ann N Y Acad Sci* **997**, 322 (Nov, 2003).
20. T. Tsilchorozidou, C. Overton, G. S. Conway, *Clin Endocrinol* **60**, 1 (January 01, 2004, 2004).
21. S. Wild, T. Pierpoint, M. P., H. Jacobs, *Clin Endocrinol* **52**, 595 (2000).
22. C. D. Gatzka, J. D. Cameron, B. A. Kingwell, A. M. Dart, *Hypertension* **32**, 575 (September 1, 1998, 1998).
23. B. A. Kingwell, *Clin Exp Pharmacol Physiol* **29**, 214 (March 01, 2002, 2002).
24. B. H. Goodpaster, D. E. Kelley, F. L. Thaete, J. He, R. Ross, *J Appl Physiol* **89**, 104 (2000).
25. B. H. Goodpaster *et al.*, *Diabetes Care* **26**, 372 (2003).
26. D. E. Kelley *et al.*, *J Clin Endocrinol Metab.* **86**, 5412 (2001).
27. S. Jung Lee, I. Janssen, S. B. Heymsfield, R. Ross, *Am J Clin Nutr* **80**, 1215 (2004).
28. C. R. Bruce, A. D. Kriketos, G. J. Cooney, J. A. Hawley, *Diabetologia* **47**, 23 (2004).
29. F. Faul, E. Erdfelder. (Bonn University, Department of Psychology, 1992).

1.15 Reporting of Results

(a) Are there any limitations or restrictions on the publication of results by researchers?

Yes ☐ No ☒

If *Yes*, explain the nature of the limitations or restrictions.

(b) Will a report of the project outcomes (for example, group data) be publicly accessible at the end of the project?

Yes ☒ No ☐

If *Yes*, give details of the type of report and how it will be made available.

If *No*, explain why not.

The results of this study will be used for a number of scientific peer-reviewed publications. Furthermore if the results prove significant, a number of publications in lay press may result

(c) Will a plain English summary of the project outcomes (for example, individual or group data) be made directly available to participants at the end of the project?

Yes ☒ No ☐ N/A ☐

If *Yes*, give details of the type of report and how it will be made available.

If *No*, explain why not.

Each participant will be provided with a confidential individual report detailing personal results in the context of the group results. They will also be invited to a post study debriefing session which will include a lecture where all group results (de-indentified mean data) will be presented in a simplified public lecture format.

1.16 Adverse or Unforeseen Events

What procedures are in place to manage, monitor and report adverse and unforeseen events? Consider adverse events in relation to all aspects of the project, including (where applicable) participants, researchers and management of information.

Participants:

Exercise and training tasks:

Exercise or physical activity is within every person's capability, and as such it is not expected to cause undue psychological distress but may be physically demanding. Any exercise task undertaken by the participants will therefore be conducted and/or prescribed on an individual basis to incorporate their capabilities. If, in the unlikely event of physical and/or psychological stress of participation in any exercise task or test the subject may immediately stop the exercise task and withdraw from the study without any prejudice. In this event the participant will be debriefed to ascertain the issues preventing their continuation with the task or test. If, after consultation with the researchers the participant is significantly distressed a counselling service will be provided at Monash University. Furthermore, there may be a small chance (<2%) of a cardiac event during any exercise testing or task, this risk is minimized by a thorough screening of the participants and all exercise tests and tasks the subjects will be monitored by using their heart rate measured with telemetry (Polar® Heart rate monitors) and not allow to exceed a rate of 180 beats per minute. If in the unlikely event of cardiac arrest during these test Dr Nigel Stepto is a trained First-aider and CPR provider and would be capable of managing such an unlikely event, and obtain medical assistance from A/Prof Ben Canny or the Campus medical staff in the Student Health services.

Cardiovascular measures:

The non invasive assessment of the vascular system has no adverse effects involves no radiation and causes no pain.

Invasive tissue sampling:
There are slight risks associated with invasive procedures in normal volunteers. These are minimised by having all procedures undertaken by a qualified and experienced medical practitioner, using accepted antiseptic techniques. The muscle and overlying fat biopsy procedure is a relatively minor surgical procedure that is rarely associated with complications such as bleeding into the muscle which causes a haematoma (blood clot). Dr Ben Canny has performed muscle biopsies in nine separate studies involving over 500 biopsies. Of these one was associated with a mild haematoma. In addition, bleeding closer to the skin surface resulting in visible bruising can also occur but has not been observed by our group. It is common to experience tenderness at the site of the muscle biopsy for 24-48 hours.
The insertion of a catheter into a forearm and hand veins by A/Prof Ben Canny, Dr Nigel Stepto (a trained phlebotomist with over 1000 successful cannulations) and/or Dimitra Giannopoulos (experienced RA with several thousand cannulations including experience in this obese population) and/or Dr Samantha Hutchison are associated with the sensation of a pin prick. There is a small chance of minor bruising as a result of insertion of the catheter. Very occasionally, however, there can be infections or clot formation due to insertion of catheters. We consider the risk extremely low (<0.05%) given the aseptic/barrier techniques used in inserting the catheter and the relatively short (<4 hours) period of time that it is in place. In the unlikely event of infections or bad bruising from either procedure, the participants will be asked to report it immediately to the researchers so that the appropriate treatment can be obtained from the clinically trained investigators.

Euglycaemic Hyperinsulineamic Clamp:
Hypoglycaemia: During these insulin clamps there is a small chance (<0.1%) of subjects/patients suffering from hypoglycaemia and associated complications. To ensure the risk of hypoglycaemic is kept to a minimum, blood glucose levels are constantly monitored in real-time (3ml blood) checked every 5 minutes using an automated blood glucose analyser, such that the glucose infusion can be matched to maintain blood glucose levels at $5mmol.L^{-1}$. In addition, well trained clinical staff (DG, SH) under the supervision of Dr Teede will be monitoring the women during the clamp.
Hypokalaemia: There is evidence that during prolonged (4h) clamps on patients (type 2 diabetics) may suffer from hypokalaemia and arrhythmia. To guard against this the patients will be administered an oral dose (30mmol) of KCL (Slow-K) at the start of the clamp. Also all diabetics are excluded from the trial at baseline. To further reduce the risks of hypokalaemia and arrhythmia the clamp duration is reduced to 2h, which when we considered all the evidence we felt we were safe in these subjects. In addition, these clamps are being conducted in the Monash University Physiology labs and a qualified clinician will be available to assist with any unforseen emergency resulting from such an intervention.

Diagnosis of an undetected condition:
There will be two distinct groups of participants, those suffering from polycystic ovarian syndrome (PCOS) and will have been previously diagnosed with the condition and be aware of the health problems and psychological stress

associated with the condition. The control subjects will be expecting to be healthy with no underlying health problems, as such all the screening tests conducted prior to the involvement in the study may detect a health problem such as PCOS, insulin resistance or diabetes mellitus and heart disease. As such the clinically trained researchers will inform the participant of this previously undiagnosed condition and assist them in obtaining the appropriate treatment and counselling. This may not exclude them from the study as long as the condition does not fall into the patient/participant exclusion criteria.

Radiation exposure:
The DEXA scan will expose the subject a minimal dose of ionising radiation of 0.1 mSv per visit. The 2 CT scans, one of the L4-L5 region of the abdomen and one of the mid-thigh will expose them to a total of 0.5 mSv per visit. Thus total ionising radiation that the volunteers will receive over the entire study is ~1.5 mSv, which is well below the annual exposure limits of 5 mSv per annum and is equivalent to 274 days of natural background radiation exposure living in Melbourne. Cancer surveys in areas of high background have failed to find increased cancer rates resulting from higher background, although Stewart did find increased childhood leukaemia among children x-rayed in utero, as such all participants will be aged 18 and over and will be tested for pregnancy if indicated in these subjects will be excluded from the study(see exclusion criteria).

Loss/theft of personal information:
In the unlikely event that personal information is lost or stolen. The subjects will be informed of this occurrence, and how it may affect them. They will also have access to counselling provided by Monash University. In addition the appropriate ethics committees will receive a report detailing the incident.

Researchers:
Radiation exposure:
When conducting the DEXA and CT scans a trained radiographer will be conducting the scans, which is a standard procedure conducted by these professionals everyday. As there training and work place safety procedures will isolate and protect them from exposure to any ionising radiation.

Human tissue and body fluid exposure:
There is a chance of researchers being exposed to human tissues and body fluids. All researchers involved are well trained and wear the appropriate protective clothing (glasses, lab coats and gloves) to minimise the risk of exposure. In the unlikely event of a needle stick injury or exposure to these tissues, there are a number of first aid and post injury management procedures in place which include wound/exposure treatment, blood testing and counselling. In addition these events are reported to the appropriate committees and organisations (MUSCEHR, SHHEC & OHSE).

In all the above cases the researchers, participates and associated people are requested to report any and all unforseen adverse events to the both the Monash University and Southern Health Human Research Ethics committees.

SECTION D: PARTICIPANTS

Researchers should consult the Guidelines under Section D for a definition of "participant" for the purposes of this application.

If the project does NOT involve participants, do NOT complete this section, but go directly to Section E. If you are not completing Section D, you may delete it from your application to avoid unnecessary paper usage.

1.17 Number of participants

(a) Total number of participants in the project (at all sites combined)

60

(b) Break down the number of participants for each site for which this HREC is responsible

Site	No. of participants
Monash University; Physiology Department; Exercise Physiology Laboratory	60
Monash Medical Centre Clayton;	60
Dandenong Hospital;	60

(c) If the project involves more than one project group (e.g. control and experimental groups), how many participants will be in each group?

Experimental groups who have PCOS and undertake exercise training:

group 1- n=group 1- Endurance training n=30

Control groups who do not have PCOS and are matched for body mass index:

group 2- Endurance exercise n=30

1.18 Participants - Details

(a) What categories of people will be recruited? *(e.g. cancer patients, children, people with learning disabilities, pensioners, etc)*

Premenopausal women with and without PCOS

(b) Will Aboriginal and Torres Strait Islander people be targeted for recruitment to this project?

☐ Yes ☒ No

If *No*, are people of Aboriginal and Torres Strait Islander origin likely to be significantly represented in the cohort of participants recruited?

☐ Yes ☒ No

(c) What will be the age range of participants?

18-40 years

(d) What ethical issues do the criteria for inclusion or exclusion give rise to?

None that is immediately apparent.

1.19 Recruitment of Participants

(a) Describe the procedure for recruitment of participants. Include information about

- Source of participants
- Exactly how potential participants will be identified
- Exactly how potential participants will be contacted and by whom, including whether the person making initial contact has any relationship to potential participants
- The method(s) by which information is provided to potential participants (*e.g. verbally, information sheet, fliers, posters, etc*)
- The setting in which information is provided (*e.g. over the telephone, in a clinic or doctor's surgery, through the mail, etc*)

Source of participants will include pre-diagnosed patients with PCOS and women in the community without condition. Participants will be made aware of the study with advertisements in the local papers, Monash University global emails, fliers and posters in the Dandenong, MMCC hospitals and Gyneacological clinics (Dr Beverley Vollenhoven). We will contact 20 controls from a previous study with an invitation to participate in this new study. If interested they can contact us. Clinicians will not directly recruit those whom they are involved in clinical care. Information will be provided in the waiting rooms with contact numbers to Dr Stepto/Dr Hutchison. The participants if interested will contact Dr Nigel Stepto who will provide a verbal explanation of the project and set up an initial meeting to provide written explanations and consent forms for their participation.

(b) Will any follow-up procedures be used to improve the rate of participation?

Yes ☐ No ☒

If *Yes*, describe the procedures.

(c) Will any dependent or unequal relationship exist between anyone involved in the recruitment and the potential participants (e.g. counsellor/client, teacher/student, doctor/patient, warder/prisoner, etc)?

Yes ☒ No ☐

If *Yes*:

(i) What is the nature of the dependent or unequal relationship?

Doctor/patient relationship

(ii) How will ethical issues arising from the unequal relationship be addressed?

There is not expected to be any ethical issues, as the doctor will not actively recruit participants. However, if the patient requests any further information from the doctor about the study, they will provide the information they have on the study and contact details for Dr Nigel Stepto/Dr Hutchison. This will in no way influence the treatment provided to the patient whether they participate or not.

(d) Will a dual relationship exist between any researcher and participants (e.g. will any of the researchers also be responsible for project, program or administrative oversight within the organisation where it is proposed to recruit participants and carry out the research)?

Yes ☐ No ☒

If *Yes*:

(i) What is the nature of the dual relationship?

(ii) How will ethical issues arising from the dual relationship be addressed?

(e) Will reimbursement, payment or other offers be made to participants?

Yes ☒ No ☐

If *Yes*, provide details.

The subjects will receive a full health check including professional exercise testing, free gym and exercise equipment usage. During the training phase of the study they will also be receiving a full personal training service to supervise exercise regimes.

1.20 Information to Participants

(a) Does the project design involve deliberate deception of participants?

Yes ☐ No ☒

If *Yes*, explain why the real purpose of the research needs to be concealed.

(b) Will information about the project be given to participants in the form of a **written** Participant Information?

Yes ☒ No ☐

If *No*, give reasons.

1.21 Consent

(a) Will any of the participants have the capacity to give voluntary and informed consent? Yes ☒ No ☐

If *Yes*, how will consent be obtained?

☒ Written consent form

☐ Verbal – explain below how consent will be recorded

☐ Implied consent (*e.g. by completing a questionnaire*) – give details

(b) Will any of the participants **not** have the capacity to give voluntary and informed consent? Yes ☐ No ☒

If *Yes*, who will be asked to provide consent?

☐ Parent/guardian

☐ Victorian Civil and Administrative Tribunal (VCAT)

☐ Other – give details

How will consent be obtained?

☐ Written consent form

☐ Verbal – explain below how consent will be recorded

(c) How will competence to give consent be determined and who will make this determination?

ATTACH A COPY OF PARTICIPANT INFORMATION AND CONSENT FORM(S) AT THE END OF MODULE ONE.

1.22 Consequences of Participation

(a) What are the potential or actual harms of participation (if any)?

There no perceived harms but the participants will be exposed to exercise tests and programmes which require physical and mental exertion, also have muscle and blood samples taken which carry small risks of bruising and infection. They will also have the infusion of sterile glucose and insulin solutions, which caries the potential risk of hypokalaemia, hypoglycaemia and arrhythmias. In addition they will be exposed to minimal doses of radiation from the 2 DEXA scans and 4 CT scouts scans.

(b) Is there any possibility of inconvenience to participants?

Yes ☒ No ☐

If *Yes*, please describe.

During the study and three months prior to any analytical procedures the subjects are required to cease insulin sensitising medication. Furthermore, the participants will be asked to switch from the oral contraceptive pill to barrier

contraception for the same duration. They will need to exercise and complete end-point measurement as described.

Exercise testing and training will require physical and mental exertion and a time commitment. We, however, consider these interventions as part of normal living and will not be to aversive for the participants to complete.

Blood sampling does carry a small discomfort, no more than experienced when having basic medical treatment and/or check-ups. Muscle sampling is considered a minor surgical procedure and does involve minor discomfort during the procedure, and may be slightly painful for 24-48h after the biopsy is taken.

The euglycaemic hyperinsulineamic clamp, is a long procedure and require the subject to remain in a supine on a bed for several hours where human insulin and a glucose solution is infused via an indwelling cannula into a forearm vein, and blood will be sampled from the opposite arm via a second cannula.

(c) Is there a need for special counselling?

Yes ☒ No ☐

If *Yes*, describe the form of the counselling: how it will be conducted, when and by whom?

During the study the subjects are required to cease insulin sensitising medication three months prior to and for the duration of the study. Furthermore the participants will be asked to switch from the oral contraceptive pill to barrier contraception over this period. This switch in contraceptive technique requires specialized counselling, which will be provided at the time of screening by AR2 & 3 (Drs Helena Teede, Beverley Vollenhoven & Samantha Hutchison).

(d) Will participants be denied access to other treatments, therapies or services as a result of participation? Yes ☒ No ☐ N/A ☐

Give details.

The participants will be required to cease using insulin sensitising medication and change contraception to barrier methods. The use of these medications (eg metformin and oral contraceptives) has profound effects on the outcome measures of this study. Thus for scientific validity and integrity it is preferable that these alternative treatments for PCOS are not administered for the duration of this study.

(e) Are there any potential benefits to the participants?

The participants will have access to professional exercise testing and prescription services. They will also gain the benefits of increased physical activity, like weight-loss, increased energy and improved lifestyles for health. Furthermore, they will undergo an extensive medical screening. During the 3 month lead in to the study they will have a number of information sessions for healthy eating, women’s health issues and the role of exercise in a healthy lifestyle.

1.23 Other Ethical Issues

Does the project present any other ethical issues with respect to participation? *(e.g. Issues related to illegal activities; indigenous or other special community or cultural groups; risks to third parties, collectivities; etc)*

N/A

SECTION E: COLLECTION/USE/DISCLOSURE OF INFORMATION

Researchers have a legal as well as an ethical obligation to consider privacy issues. The following questions assist both the researcher and the HREC to fulfil their obligations under State and Commonwealth privacy legislation.

You may delete questions or parts of questions that you are not required to answer, in the interests of reducing paper usage.

1.24 Collection of Information Directly from Individuals

(a) Does the project involve collection of information directly from individuals about themselves?

☐ No - **go to Question 1.25**

☒ Yes – answer the following questions:

(b) What type of information will be collected? (*Tick as many as apply*)

☒ personal information

☒ sensitive information

☒ health information

(c) Does the Participant Information and Consent Form explain the following:

The identity of the organisation collecting the information and how to contact it?	Yes☒ No☐
The purposes for which the information is being collected?	Yes☒ No☐
The period for which the records relating to the participant will be kept?	Yes☒ No☐
The steps taken to ensure confidentiality and secure storage of data?	Yes☒ No☐
The types of individuals or organisations to which your organisation usually discloses information of this kind?	Yes☒ No☐
How privacy will be protected in any publication of the information?	Yes☒ No☐
The fact that the individual may access that information?	Yes☒ No☐
Any law that requires the particular information to be collected?	Yes☐ No☒
The consequences (if any) for the individual if all or part of the information is not provided	Yes☒ No☐

If you answered "*No*" to any of these questions, give the reasons why this information has not been included in the Participant Information and Consent Form.

At this time we are unaware of any law or situation applicable to this study population that would require us to collect any personal information sensitive or otherwise.

1.25 Do Other Questions in this Section have to be Completed?

(a) Does the project involve the collection, use or disclosure of **identified or potentially identifiable** information from sources other than the individual whose information it is? *(see Module One Guidelines for definitions)*

☐ No – **Go to Question 1.30 and do not answer the remainder of question 1.25, 1.26, 1.27, 1.28 or 1.29**

☒ Yes – **answer the following question**

(b) Does the project involve the collection, use or disclosure of information **without the consent** of the individual whose information it is (or their legal guardian)?

☒ No – **Go to Question 1.30 and do not answer questions 1.26, 1.27, 1.28 or 1.29**

☐ Yes – **answer the following questions**

1.26 Type of Activity Proposed

Are you seeking approval from this HREC for

(a) collection of information from a third party?

☐ Yes – **start at Question 1.27**

☐ No – **start at Question 1.30**

(b) use of information?

☐ Yes ☐ No

(c) disclosure of information?

☐ Yes ☐ No

1.27 Collection of Information from a Third Party

Only answer this question if the project involves the collection of identified (or potentially identifiable) information from a source other than the individual (or their legal guardian) without the consent of the individual or their legal guardian.

(a) From which of the following sources will information be collected? *(Tick as many as apply)*

	**Source of Information**
☐	A Victorian public health service provider
☐	A Victorian private health service provider
☐	An organisation other than a health service provider
☐	A data set under the auspices of the Victorian DHS
☐	A data set under the auspices of another Victorian

	government department
☐	A data set from another Victorian source
☐	A Commonwealth agency
☐	An agency from another state
☐	An "organisation" as defined in s95A of the Privacy Act
☐	An individual (such as a carer)
☐	Other

List the categories of individuals or organisations from which information will be collected. If information will be collected from more than one category, indicate clearly what information or records will be collected from each category.

**Category**	**Type of information or records to be collected**
e.g. carers; hospitals	*e.g. contact information; complete medical history*

(b) Have all organisations from which the information is to be collected agreed to provide the information or to allow access to the information?

☐ Yes ☐ No

If *Yes*, provide evidence of this agreement. Provide details of any conditions imposed by the organisation(s) concerning the release of the information.

If *No*, explain how and when the agreement of the disclosing organisation will be obtained.

(c) Is any organisation from which the information will be collected seeking separate HREC approval for disclosure of the information? *(See the Module One Guidelines for further explanation of this question. Note: The organisation(s) disclosing the information is not required by law to obtain separate HREC approval to disclose the information. However, some institutions may wish to obtain separate approval for disclosure for their own purposes.)*

☐ Yes – supply a copy of the decision from the other HREC (when available)

☐ No - a copy of any approval from this HREC will have to be forwarded to the disclosing organisation

(d) Does the person who is collecting the information routinely have access to that information?

☐ Yes ☐ No

(e) What information will be collected? (*Tick all boxes that apply*)

	Type of information		Type of organisation(s) involved	Privacy Principle(s)
☐	Health information	☐	Victorian public sector	HPP 1
		☐	Victorian private sector	HPP 1, NPP 1, NPP 10
		☐	Commonwealth public sector	IPP 11
		☐	Other	NPP 1, NPP 10
☐	Personal information (other than health information)	☐	Victorian public sector	VIPP 1
		☐	Victorian private sector	NPP 1
		☐	Commonwealth public sector	IPP 11
		☐	Other	NPP 1
☐	Sensitive information	☐	Victorian public sector	VIPP 10
		☐	Victorian private sector	NPP 10
		☐	Commonwealth public sector	IPP 11
		☐	Other	NPP 10

(f) Give reasons why information will not be collected in a de-identified form.

(g) For what reason(s) will consent not be obtained from the individual(s) whose information will be collected?

(h) Give reasons why the proposed collection of information is in the public interest. Note that the public interest in the proposed research must substantially outweigh the public interest in respecting individual privacy.

1.28 Use of Information

Only answer this question if the project involves the use of identified (or potentially identifiable) information without the consent of the individual whose information it is (or their legal guardian).

(a) What information will be used? (*Tick all boxes that apply*)

	Type of information		Type of organisation(s) involved	Privacy Principle(s)
☐	Health information	☐	Victorian public sector	HPP 2
		☐	Victorian private sector	HPP 2, NPP 2
		☐	Commonwealth public sector	IPP 11
		☐	Other	NPP 2
☐	Personal information (other than health information)	☐	Victorian public sector	VIPP 2
		☐	Victorian private sector	NPP 2
		☐	Commonwealth public sector	IPP 11
		☐	Other	NPP 2
☐	Sensitive information	☐	Victorian public sector	VIPP 2
		☐	Victorian private sector	NPP 2
		☐	Commonwealth public sector	IPP 11
		☐	Other	NPP 2

(b) What are the specific purposes for which the information will be used?

(c) Is the purpose for which the information will be used (the secondary purpose) related to the purpose for which the information was **originally** collected (the primary purpose)?

☐ Yes ☐ No

Give details.

(d) Give reasons why information will not be used in a de-identified form. *(If the answer is the same as for Q1.27 (f), write "as above".)*

(e) For what reason(s) will consent not be obtained from the individual(s) whose information will be used? *(If the answer is the same as for Q1.27 (g), write "as above".)*

(f) Give reasons why the proposed use of information is in the public interest. Note that the public interest in the proposed research must substantially outweigh the public interest in respecting individual privacy. *(If the answer is the same as for Q1.27 (h), write "as above".)*

1.29 Disclosure of Information

Only answer this question if the project involves the disclosure of identified (or potentially identifiable) information without the consent of the individual whose information it is (or their legal guardian).

(a) Will identified (or potentially identifiable) information be disclosed by an organisation to the researcher?

☐ No – **Go to question 1.29(b)**

☐ Yes – answer the following question

What information will be disclosed by the organisation(s) to the researcher? (*Tick all boxes that apply*)

	Type of information		Type of organisation(s) involved	Privacy Principle(s)
☐	Health information	☐	Victorian public sector	HPP 2
		☐	Victorian private sector	HPP 2, NPP 2
		☐	Commonwealth public sector	IPP 11
		☐	Other	NPP 2
☐	Personal information (other than health information)	☐	Victorian public sector	VIPP 2
		☐	Victorian private sector	NPP 2
		☐	Commonwealth public sector	IPP 11
		☐	Other	NPP 2
☐	Sensitive information	☐	Victorian public sector	VIPP 2
		☐	Victorian private sector	NPP 2
		☐	Commonwealth public sector	IPP 11
		☐	Other	NPP 2

List the organisations that will disclose information to the researcher. If more than one organisation is involved, indicate clearly what information or records will be disclosed by each organisation to the researcher.

(b) Will identified (or potentially identifiable) information be disclosed by the researcher to other organisations?

☐ No – **Go to question 1.30**

☐ Yes – answer the following questions

What information will be disclosed by the researcher? (*Tick all boxes that apply*)

	Type of information		Type of organisation(s) involved	Privacy Principle(s)
☐	Health information	☐	Victorian public sector	HPP 2
		☐	Victorian private sector	HPP 2, NPP 2
		☐	Commonwealth public sector	IPP 11
		☐	Other	NPP 2
☐	Personal information (other than health information)	☐	Victorian public sector	VIPP 2
		☐	Victorian private sector	NPP 2
		☐	Commonwealth public sector	IPP 11
		☐	Other	NPP 2
☐	Sensitive information	☐	Victorian public sector	VIPP 2
		☐	Victorian private sector	NPP 2
		☐	Commonwealth public sector	IPP 11
		☐	Other	NPP 2

List the organisations to which information will be disclosed. If information will be disclosed to more than one organisation, indicate clearly what information or records will be disclosed in each case.

(c) Give reasons why information will not be disclosed in a de-identified form. *(If the answer is the same as for Q1.27 (f) or Q1.28 (d), write "as above".)*

(d) For what reason(s) will consent not be obtained from the individual(s) whose information will be disclosed? *(If the answer is the same as for Q1.27 (g) or Q1.28 (e), write "as above".)*

(e) Give reasons why the proposed disclosure of information is in the public interest. Note that the public interest in the proposed research must substantially outweigh the public interest in respecting individual privacy. *(If the answer is the same as for Q1.27 (h) or Q1.28 (f), write "as above".)*

1.30 General Issues

(a) How many records will be collected, used or disclosed? Specify the information that will be collected, used or disclosed *(e.g. date of birth, medical history, number of convictions, etc)*

Number of records: 4
Type of information:
Name and contact information
Date of Birth
body measures and composition
medical history
Training diaries
Menstrual diaries

14 day food diaries

(b) Does the project involve the adoption of unique identifiers assigned to individuals by other agencies or organisations?

☐ Yes ☒ No

If *Yes*, give details of how this will be carried out in accordance with relevant Privacy Principles (e.g. HPP 7, VIPP 7 or NPP 7).

(c) Does the project involve trans-border (i.e. interstate or overseas) data flow?

☐ Yes ☒ No

If *Yes*, give details of how this will be carried out in accordance with relevant Privacy Principles (e.g. HPP 9, VIPP 9 or NPP 9).

(d) For what period of time will the information be retained? How will the information be disposed of at the end of this period?

The information will be stored in the Physiology Department for 5 years. It will then be destroyed by a shredding, conducted by a certified company used by the University to destroy other confidential printed material.

(e) Describe the security arrangements for storage of the information. Where will the information be stored? Who will have access to the information?

The information will be stored in the Exercise Physiology Laboratory under lock and key. The only people who will have access to the data are the researchers involved in the study. The individual will have access to their individual data upon request.

(f) How will the privacy of individuals be respected in any publication arising from this project?

Any publications will have data presented as means, as such no individual can be identified.

1.31 Other Ethical Issues

Discuss any other ethical issues **relevant to the collection, use or disclosure of information** proposed in this project. Explain how these issues have been addressed.

SECTION F: FINANCIAL AND RELATED ISSUES

1.32 Potential Conflict of Interest

Do any researchers have any financial interests in this research or its outcomes, or any relevant affiliations?

Yes ☐ No ☒

If *Yes*, give details

If you have declared a potential conflict of interest, you should include an appropriate comment on the Participant Information and Consent Form.

1.33 Indirect Costs

Will there be payments over and above the direct costs of this project (e.g. conference and travel, recruitment incentives, equipment)?

Yes ☐ No ☒

If *Yes*, please provide details of payments and justification for them.

1.34 Project Budget

Attach a detailed project budget to this application.

Have you included:

- Salaries with on-costs ☒
- Administration costs ☐
- Research consumables (for example, bed-day costs) ☒
- Participant reimbursement ☐
- Departmental charges (e.g. Pharmacy, Pathology, Radiology) ☒

If a detailed budget is not being provided, give reasons.

Salary costs:

0.16 RA (Level A 3 with 15.35% on costs 41 663) $ 6 666

Consumables:

DEXA and CT scan analysis of Body Composition (120 tests) $20 400

Exercise testsing (120 tests)	$ 2 400
Insulin clamps (120 tests)	$12 000
Blood analysis:	
glucose/lactate (1440 samples)	$ 2 880
Muscle and fat analysis:	
Protein extraction and analysis	$ 8 000
mRNA extraction and expression analysis	$12 000
**Pathology services for blood analysis:**	
Lipid profiles (120 samples)	$ 2 280
HbA_{1C} (60)	$ 849
Insulin analysis (840 samples)	$18 774
Androgen analysis (120 samples)	$ 1 956
Progesterone (120 samples)	$ 1 956
Oestrogen (120 samples)	$ 1 956
SHBG (120 samples)	$ 1 956

1.35 Source of Funding

How will this project be funded? List all sources of funds (*e.g. commercial sponsorship, grant, departmental funds etc*).

		**Status of Funds**	
**Source**	**Amount in $**	**Application pending**	**Funds Available**
Monash University Strategic Grants	35 000		Available
Departmental Funding	30 000		Available

1.36 Funds Coverage

Do the funds presently available or applied for cover all requirements to conduct the project?

Yes ☐ No ☒

If *No*, explain how the shortfall will be made up or dealt with.

The shortfall of funds is hoped to be met with additional funding from new grant applications to NH & MRC. The current funding available however will allow the on half of the project to run, investigating one exercise mode (endurance training). If the funding can not be met the study in the current form will then cease to run.

1.37 Claims through Medicare

Will any charges be incurred by Medicare as a result of patient screening or

participation?

Yes ☐ No ☒ N/A ☐

If *Yes*, has the Health Insurance Commission been notified and have they given permission?

Yes ☐ No ☐

1.38 Declaration by Researchers

Project Title: Role of exercise in treatment of women with polycystic ovary syndrome: Mechanisms of action.

I/WE, the researcher(s) agree:

- To only start this research project after obtaining final approval from the Institution's Human Research Ethics Committee (HREC);
- To only carry out this research project where adequate funding is available to enable the project to be carried out according to good research practice and in an ethical manner;
- To provide additional information as requested by the HREC;
- To provide progress reports to the HREC as requested, including a final report and a copy of any published material at the end of the research project;
- To maintain the confidentiality of all data collected from or about project participants;
- To notify the HREC in writing immediately if any change to the project is proposed and await approval before proceeding with the proposed change;
- To notify the HREC in writing immediately if any adverse event occurs after the approval of the HREC has been obtained;
- To agree to an audit if requested by the HREC;
- To only use data and any tissue samples collected for the study for which approval has been given;
- To only grant access to data to authorised persons; and
- To maintain security procedures for the protection of privacy, including (but not restricted to): removal of identifying information from data collection forms and computer files, storage of linkage codes in a locked cabinet and password control for access to identified data on computer files.

I/we have read the NH&MRC *National Statement on Ethical Conduct in Research Involving Humans* 1999 and will observe the principles set out in that document and in the *Declaration of Helsinki*.

Name of principal researcher …Nigel Stepto………………………………………………………….

Signature Date

Name of researcher …………Helena Teede……………………………………………….

Signature Date

Name of researcher …………Beverley Vollenhoven………………………………………………..

Signature Date

Name of researcher ....Benedict Canny...

Signature Date

Name of researcher ....Samantha Hutchison...

Signature Date

Name of researcher ....Cheryce Harrison...

Signature Date

Name of researcher ....Lisa Moran...

Signature Date

Name of researcher .... Dominic Rachon ....

Signature Date

1.39 Certification by Principal Researcher and Head of Department

Project Title: Role of exercise in treatment of women with polycystic ovary syndrome: Mechanisms of action.

Certification By Principal Researcher

I accept responsibility for the conduct of this research project according to the principles of the *National Statement on Ethical Conduct in Research Involving Humans* published by the National Health & Medical Research Council (June 1999).

I certify that all researchers and other personnel involved in this project are appropriately qualified and experienced or will undergo appropriate training to fulfil their role in this project.

As principal researcher, I will take responsibility for the confidential maintenance of records for 7 years after completion of the project (15 years in the case of drug trials).

Name of principal researcher: ......Nigel Stepto......................................

Signature Date 12-10-2007

Acceptance by Head of Department

I certify that I have read the research project application named above.

My signature indicates that I support this research project.

Name of Head of Department: ……………………………………

Name of Department: ……………………………………

Signature   Date

1.40 Declaration by Head of Supporting Department

This form is to be completed by the Head of any Department that is providing support or services to the research project, but which does not have any member(s) on the research team.

If completing this form by hand, please use BLACK INK only.

**Project Title:** Role of exercise in treatment of women with polycystic ovary syndrome: Mechanisms of action.<br>**Principal Researcher: Dr Nigel Stepto**

I have discussed this project with the Principal Investigator and have seen the application and protocol. I am (*tick whichever applies*)

☐ able to perform the investigations/services indicated, within the present resources of the Department;

☐ able to perform the investigations/services indicated, if the following financial assistance is provided:

☐ unable to undertake the investigations/services indicated, on the following grounds:

Name: ……………………………………………………………….

Signature: ……………………………………………….. Date:

Head of the Department of ………………………………………………

MODULE ONE: CHECKLIST

Please satisfy each of the following before submitting the application. Failure to do so will delay review of the application.

Include a copy of this checklist (completed & signed) with the application.

Full Project Title

Role of exercise in treatment of women with polycystic ovary syndrome: Mechanisms of action.

Have you answered all relevant questions in Module 1?	☐
Is a staff member from the Institution listed as a co-researcher?	☐
Have you defined all technical terms and abbreviations used?	☐
Have you included all questionnaires or surveys to be used?	☐
Have you completed all financial details in Module 1, Section F?	☐
Have you included a detailed project budget?	☐
Have you declared all potential conflicts of interest?	☐
Have you included any other site-specific modules or documentation specifically required by the Institution(s) at which you intend to conduct your research?	☐
Do the Participant Information and Consent Form(s) show the name of the Institution, with pages numbered & dated in the footer?	☐
Are all relevant modules stapled separately, in order? *Note: Attach attachments for each module at the end of that module*	☐
Are all pages (including attachments) numbered in the footer?	☐
Have you provided an original and the required number of copies?	☐
Have you completed the form "Declaration by Researcher(s)?	☐
Have you completed the form "Certification by Principal Researcher and Head of Department"?	☐
Has a completed "Declaration by Head of Supporting Department" been included for each supporting department (if applicable)?	☐

Name of principal researcher-…………………………………….

Signature Date
